# Development of new ruthenium complex-based photocatalysts for the degradation of Remazol Red Ultra RGB

**DOI:** 10.1039/d6ra06712c

**Published:** 2026-10-28

**Authors:** Dayane C. de Oliveira, Diana T. de L. Souza, Wesley E. C. Cavalcanti, Johnatan de O. Soares, Daniel de L. Pontes, Ana C. F. de B. Pontes, Tércio de F. Paulo, Sibele B. C. Pergher, e Francisco O. N. da Silva

**Affiliations:** a Coordination Chemistry and Polymers Laboratory (LQCPol), Institute of Chemistry, Federal University of Rio Grande do Norte (UFRN) Natal 59078-970 Brazil dayane.campelo.702@ufrn.edu.br; b Bioinorganic Laboratory, Department of Organic and Inorganic Chemistry, Federal University of Ceará (UFC) P.O. Box 6021 Fortaleza CE 60440-900 Brazil; c Molecular Sieves Laboratory (LABPEMOL), Institute of Chemistry, Federal University of Rio Grande do Norte (UFRN) Natal 59078-970 Brazil

## Abstract

The contamination of aquatic environments by persistent synthetic dyes constitutes a significant environmental problem, with Remazol Red Ultra RGB standing out due to its high chemical stability and wide industrial application. In this work, ruthenium-based complexes were developed and evaluated as photocatalysts for the degradation of this dye in aqueous medium. The complexes were characterized by UV-Vis and infrared spectroscopy, cyclic voltammetry, and theoretical studies based on DFT and TD-DFT. In the photocatalytic assays, the materials showed high degradation rates, with emphasis on the NAT60 complex, which promoted 100% dye removal within 60 minutes of irradiation, evidenced by the decrease in the absorption band at 514 nm. The other complexes showed efficiencies higher than 70% within the same time interval. In order to evaluate the effect of a high surface area matrix, the catalyst was supported on mesoporous silica of the SBA-15 type, forming the NAT60/SBA-15 system, which maintained an efficiency of 85.25%, demonstrating high photocatalytic activity. The results confirm the potential of ruthenium complexes, both in molecular and supported forms, as promising photocatalysts for advanced oxidative processes applied to the sustainable treatment of dye-containing effluents.

## Abstract 2

Schematic representation of the photocatalytic cycle of ruthenium complexes for Remazol Red Ultra RGB dye removal, showing dye adsorption and color removal after 60 min, with efficiencies of 75.50% (NAT41), 100% (NAT60), and 85.24% (NAT60/SBA-15).

## Graphical abstract

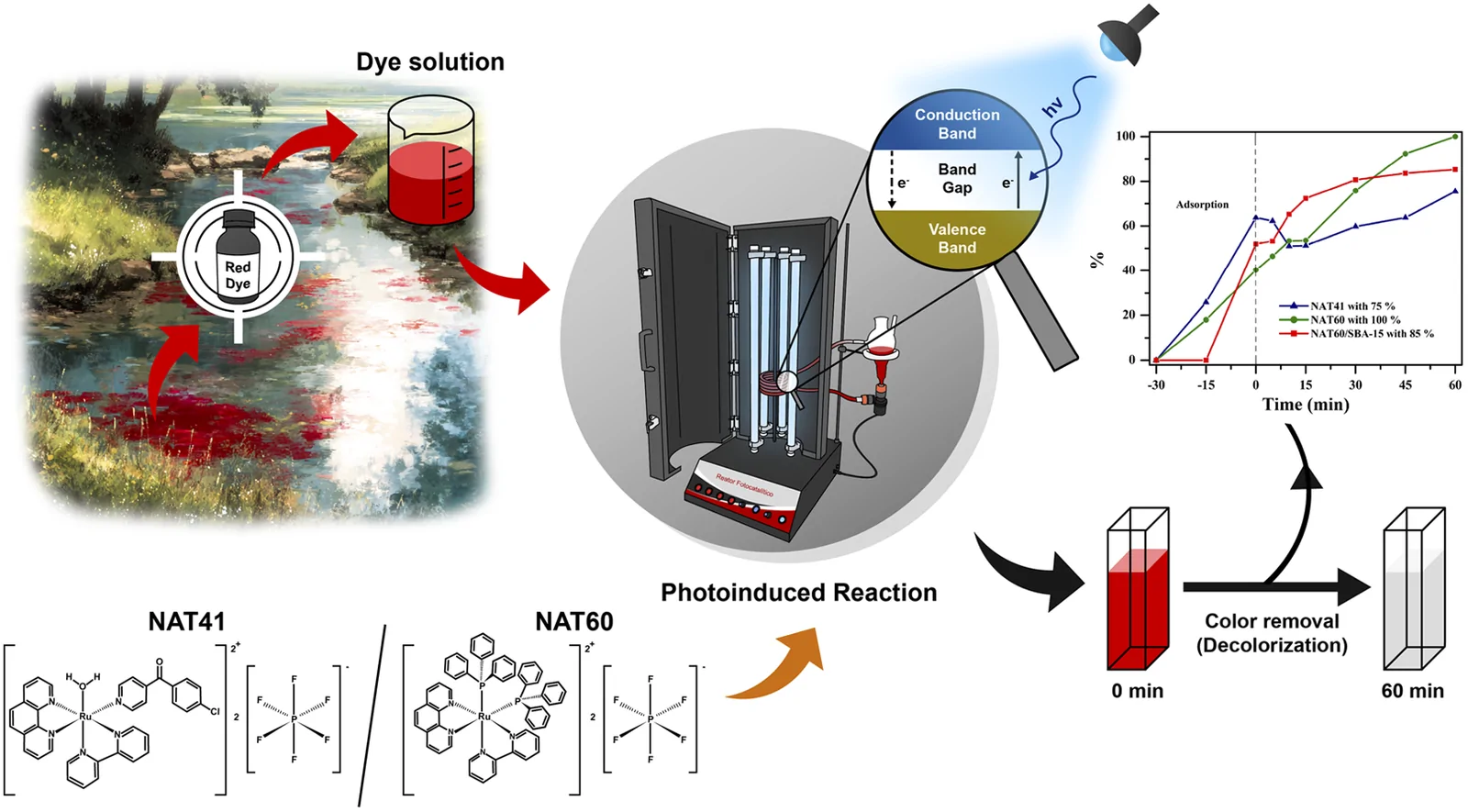

## 1. Introduction

The contamination of effluents by anthropogenic activities constitutes one of the main environmental challenges today, compromising the balance of aquatic ecosystems and the quality of water resources.^1^ Considering that aquatic environments occupy more than two-thirds of the planet's surface and play an essential role in climate regulation and the maintenance of ecosystem services,^2^ their degradation represents a global-scale threat. Among the different contamination pathways, the discharge of industrial waste stands out, being responsible for significant impacts on water quality.^3,4^ It is estimated that the industrial sector consumes approximately 20% of the freshwater available on the planet, reinforcing the urgency of adopting efficient strategies to mitigate these impacts.^5^ In the national context, CONAMA Resolution No. 430 establishes that the discharge of effluents must occur in a controlled manner, avoiding damage to the uses of water bodies.^6^

Among the most recurrent industrial contaminants are synthetic dyes, widely used in the textile, paper, leather, and plastics industries.^5,7^ It is estimated that tons of these compounds are released annually into water bodies, altering physicochemical parameters such as turbidity, light penetration, and oxygenation.^8^ Structurally, dyes are composed of chromophore groups, responsible for color and acting as electron acceptors, and auxochrome groups, which favor fixation to the substrate through electron donation.^9,10^ The structural diversity of these compounds allows them to be classified as reactive, acidic, basic, and direct dyes, among others, in addition to being subdivided into azo, triphenylmethane, heterocyclic, anthraquinone, and polymeric dyes.^11^

The high chemical stability of these compounds, resulting from extensive conjugated aromatic systems, gives them low biodegradability and high environmental persistence.^12,13^ In addition, many dyes present xenobiotic character and potential mutagenic and carcinogenic effects.^14^ Even at low concentrations, they are easily visually detectable, compromising the penetration of solar radiation in the water column, reducing photosynthesis, and negatively impacting aquatic biota.^15^

Several strategies have been employed in the treatment of dye-containing effluents, including membrane filtration,^16^ adsorption,^17^ electrocoagulation,^18^ bioaugmentation,^19^ and ozonation.^20^ In general, these methods can be classified as physical, chemical, or biological,^21^ presenting limitations related to efficiency, operational cost, or generation of secondary waste.^22^ In this context, photocatalytic processes have stood out as promising alternatives for the degradation of organic contaminants under mild conditions.^23,24^

In heterogeneous photocatalysis, the absorption of radiation with energy equal to or greater than the band gap of the material promotes the generation of electron–hole pairs (e^−^/h^+^), capable of reacting with water molecules or adsorbed hydroxyl ions, forming hydroxyl radicals (˙OH), highly oxidizing species responsible for the degradation of complex organic compounds.^25,26^ Among the advantages of this process are operation under ambient temperature and pressure, reduced need for chemical reagents, and ease of recovery of the solid catalyst.^27^

Materials based on transition metal complexes have received increasing attention due to their structural versatility and tunable photoactive properties. The advancement of organometallic chemistry, especially from the second half of the 20th century, significantly expanded the understanding of catalytic mechanisms and the application of these systems in relevant industrial processes, such as Ziegler, Wacker, and Oxo processes.^28^ Emblematic examples include Wilkinson's catalyst in olefin hydrogenation^28^ and the ruthenium complexes developed by Grubbs for metathesis reactions, whose importance was recognized with the Nobel Prize in Chemistry in 2005.^29,30^ The possibility of modulating electronic and steric effects through the appropriate choice of ligands expands the potential of these systems in sustainable catalysis applications.

Among the various commercial dyes, Remazol Red Ultra RGB stands out due to its lower reactivity and greater stability in aqueous medium, showing high resistance to degradation.^31–34^ This characteristic makes it a relevant model molecule for evaluating the efficiency of new photocatalytic systems.

In this context, the present work aims to investigate the application of ruthenium complexes, also supported on mesoporous SBA-15 matrix, in the photocatalytic degradation of the dye Remazol Red Ultra RGB under LED irradiation. The integration between coordination chemistry and heterogeneous photocatalysis seeks to contribute to the development of sustainable and efficient technologies aimed at the treatment of industrial effluents.

## 2. Methodology

### 2.1. Reagents

Hydrated ruthenium trichloride – RuCl_3_·*x*H_2_O (Sigma-Aldrich; 99%) was used as the starting material for the synthesis of the complexes. The ligands 2,2′-bipyridine, 1,10′-phenanthroline, and sodium nitrite were obtained from Sigma-Aldrich (99%), Vetec Fine Chemistry (99.5%), and Neon (99.6%), respectively, and were used without any further purification.

### 2.2. Synthesis of the compounds

The compound *cis*-[Ru(bpy)(phen)Cl_2_] was obtained according to the synthesis methodology described by Penha (2018).^35^ Electronic spectrum in methanol: 221 nm (3.5 × 10^4^ L mol^−1^ cm^−1^), 266 nm (4.3 × 10^4^ L mol^−1^ cm^−1^), 289 nm (3.0 × 10^4^ L mol^−1^ cm^−1^), 350 nm (4.6 × 10^3^ L mol^−1^ cm^−1^), 455 nm (7.2 × 10^3^ L mol^−1^ cm^−1^), and 515 nm (8.6 × 10^3^ L mol^−1^ cm^−1^). Infrared data (ATR, cm^−1^): *ν*_as_(C–H) 3057, *ν*_sym_(C–H) 3031, *ν*(C=C) 1595, 1565, *ν*(C=N) 1443, 1417, *δ*(C–H) in-plane 1147, *δ*(C–H) out-of-plane 842, 760, 716. Electrochemical data: *E*_1/2_ = 479 mV (*vs.* Ag/AgCl) for the Ru^3+^/Ru^2+^ redox couple.

### 2.3. *cis*-[Ru(bpy)(phen)(4-Clpy)H_2_O](PF_6_)_2_ – NAT41 (1)

Compound 1 was synthesized by dissolving the precursor complex *cis*-[Ru(bpy)(phen)Cl_2_] (0.100 g, 0.197 mmol) and 4-(4-chlorobenzoyl)pyridine (0.047 g, 0.217 mmol) in a water and ethanol mixture in a 1 : 1 ratio (10 mL). The reaction system was kept under stirring and reflux for 4 h at 90 °C. After this time, the volume was reduced to half until thermal equilibrium was reached. Then, ammonium hexafluorophosphate (0.15 g) was added to precipitate the complex. Finally, the obtained product was filtered and stored under vacuum, and the reaction yielded 93%. Elemental analysis calculated for C_34_H_26_ClN_5_O_2_Ru·2PF_6_. Found (calculated): C, 41.65 (42.41); H, 2.45 (2.7); N, 7.98 (7.27)%.

### 2.4. *cis*-[Ru(bpy)(phen)(PPh_3_)_2_](PF_6_)_2_ – NAT60 (2)

Compound 2 was synthesized by dissolving the precursor complex *cis*-[Ru(bpy)(phen)Cl_2_] (0.100 g, 0.197 mmol) and triphenylphosphine (0.113 g, 0.433 mmol) in water and ethanol in a 1 : 2 ratio. The reaction system was kept under stirring and reflux for 4 h at 90 °C. After this time, the volume was reduced and the solution was allowed to cool until thermal equilibrium was reached. Then, a solution of ammonium hexafluorophosphate (0.15 g) was added to precipitate the complex. Finally, the compound was filtered, washed with hexane, and stored under vacuum, and the reaction yielded 87%. Elemental analysis calculated for C_58_H_46_N_4_P_2_Ru·2PF_6_. Found (calculated): C, 57.03 (72.41); H, 3.12 (4.78); N, 4.78 (5.82)%.

### 2.5. NAT60/SBA-15 (3)

For the preparation of NAT60/SBA-15, the impregnation of compound 2 onto the mesoporous material (SBA-15) was carried out, as described by Cavalcanti (2023).^36^ The method employed was wet impregnation, using a proportion of 10% complex (compound 2) and 90% SBA-15. The synthesis started from 10 mL of the stock solution of compound 2, to which 0.9 g of SBA-15 and 2 mL of an aqueous PF_6_^−^ solution were added, and the mixture was kept under mechanical stirring for 10 min in a beaker. Subsequently, the solution was placed in an oven for 4 h at 60 °C for drying, yielding a light orange solid.

### 2.6. Instrumentation

Electronic spectra (UV-Vis) were recorded on a Shimadzu spectrophotometer, model 1800, in the range of 190 to 1000 nm, using 1 cm quartz cuvettes. The molar absorptivity coefficients (*ε*) were determined based on the Lambert–Beer law, using solutions of defined concentrations. The photocatalytic test was monitored using a Hach DR 5000 UV-Vis spectrophotometer, following the variation in the intensity of the band at 514 nm. Vibrational spectra (IR) were obtained from solid samples using an ATR crystal, within a spectral window of 600 to 4000 cm^−1^. The spectra were recorded using an infrared spectrophotometer from Agilent Technologies, Cary 630 FTIR. Electrochemical analyses (CV) were carried out using an Epsilon potentiostat (BASi, Bioanalytical Systems Inc.), through an electrochemical cell composed of three electrodes: an Ag/AgCl reference electrode, a glassy carbon working electrode, and a platinum auxiliary electrode. In addition, two electrolytes were used for the analyses: 0.1 mol L^−1^ KCl electrolyte at pH 3.6 and 0.1 mol L^−1^ NaTFA electrolyte at pH 3.5.

Density functional theory (DFT) calculations were performed using the Gaussian 16 software package, revision C.01 (Gaussian Inc., Wallingford, CT). The computational calculations were conducted using the hybrid functional B3LYP. For lighter atoms (C, H, N, O, F, and Cl), the 6-31+G(d,p) basis set was employed, while for the ruthenium atom (Ru), the LanL2DZ basis set was applied, which incorporates a relativistic effective core potential.

### 2.7. Computational details

All density functional theory (DFT) and time-dependent DFT (TD-DFT) calculations for complexes NAT41 and NAT60 were performed using the B3LYP functional as implemented in the Gaussian 16 software package, revision C.01 (Gaussian Inc., Wallingford, CT).^37^ The 6-31+g(d,p) basis set was employed for non-metallic atoms, while the LANL2DZ basis set was used to describe metallic centers. Vibrational analyses were carried out following each geometry optimization, and the absence of imaginary frequencies confirmed that the structures correspond to true minimum on the potential energy surface. Vibrational transitions were obtained from geometries optimized in the gas phase, whereas for electronic transitions the complexes were optimized including solvent effects (methanol) through Integral Equation Formalism Polarizable Continuum Model (IEFPCM). Data were extracted from the output files using GaussSum,^38^ and natural transition orbitals (NTOs) were obtained using the Multiwfn v3.8 software^39^ package.

## 3. Results and discussion

### 3.1. Complex characterization

The characterization of complexes NAT41 and NAT60 was carried out using infrared and UV-Vis spectroscopies, as well as cyclic voltammetry techniques complemented by DFT calculations to help in the assignments of vibrational and electronic transitions.

Fig. S1 and S2 present the experimental and calculated IR spectra of the NAT41 and NAT60 complexes, respectively. There is a notable correlation between computed and experimental IR spectral profiles. Therefore, the computed data are precise enough to help with the assignment of main vibrational modes for both complexes (see Table S1).

For NAT41 (Fig. S1), the band observed at 1662 cm^−1^ is attributed to the carbonyl bond stretching, *ν*(CO), of the 4-chlorophenyl-(pyridin-4-yl)methanone (4-Clpy) ligand. The bands observed at 1340, 1280, 1089, and 935 cm^−1^ correspond to various *ν*(CC) stretching vibrational modes of 4-Clpy as detailed in Table S1. For the phen ligand, the band at 1518 cm^−1^ corresponds to the *ν*(CC) stretching mode. The band at 1427 cm^−1^ corresponds to CH symmetric bending modes in-plane, *ρ*(CH), while the bands at 878 and 718 cm^−1^ are associated with CH symmetric bending modes out-of-plane, *ω*(CH). Additionally, the bands at 1402 and 761 cm^−1^ correspond to the *ρ*(CH) and *ω*(CH) modes of bpy, respectively.

For NAT60, the bands at 1481 and 1429 cm^−1^ correspond to the *ρ*(CH) mode, whereas the band at 750 cm^−1^ is associated with the *ω*(CH) mode of the phenyl (Ph) rings of the triphenylphosphine (PPh_3_) ligand. Furthermore, the bands at 1091 and 999 cm^−1^ correspond to the breathing modes of the phenyl rings. The bands at 1631 and 761 cm^−1^ are assigned to the *ν*(CC) and *ω*(CH) modes of the bpy ligand, respectively. Regarding the vibrational modes of the phen ligand, the bands exhibit very low intensity or are superimposed by the strong band of the counter-ion at 832 cm^−1^.

Therefore, the agreement between the calculated and experimental spectra supports the reliability of the proposed structures for complexes NAT41 and NAT 60.

Cyclic voltammetry (CV) was employed to investigate the electrochemical behavior of the complexes, as shown in Fig. 1. A reversible redox process with *E*_1/2_ values of 709 (NAT41) and 0.806 V (NAT60) assigned to the Ru^3+^/Ru^2+^ pair. The π-acceptor character of the 4-(4-chlorobenzoyl)pyridine and triphenylphosphine (PPh_3_) ligands leads to a reduction in electron density at the metal center, promoting the oxidation process of the complex to occur at a more positive potential compared to the [Ru(bpy)(phen)Cl_2_] precursor (*E*_1/2_ = 0.356 V). Besides, the bidentate Pph_3_, in comparison to the OH_2_ and 4-Clpy ligands, enhanced the stabilization of the metallic center in its reduced state.

**Fig. 1:**
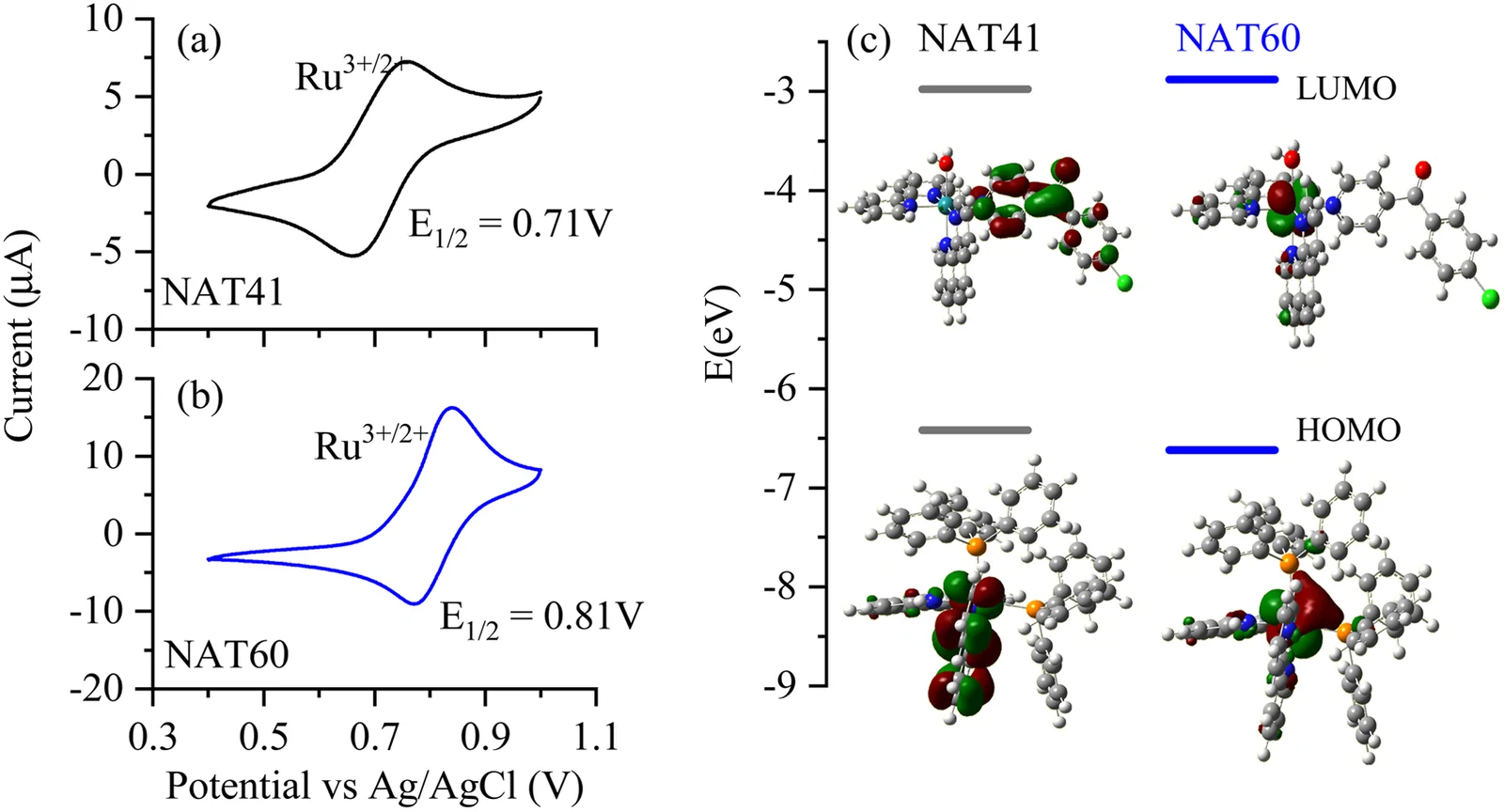
Cyclic voltammograms at 0.1 V s^−1^ of glass carbon electrode in aqueous solution containing (a) NAT41 and (b) NAT60 (c) energy level diagram of HOMO and LUMO. Inset: HOMO and LUMO isosurfaces (isovalue 0.04).

The distribution of frontier molecular orbitals from DFT calculations indicates that the lowest unoccupied molecular orbitals (LUMO) are predominantly located on the 4-Clpy and phen ligands for NAT41 and NAT60, respectively. In contrast, the highest occupied molecular orbitals (HOMO) are primarily situated on the metal center, as shown in figure. Furthermore, DFT calculations suggest that the HOMO (Fig. 2(c)) of the complexes is mainly centered on the metal, with the HOMO of NAT60 being 0.2 eV more negative than that of NAT41. Therefore, the computed data were consistent with experimental findings.

**Fig. 2:**
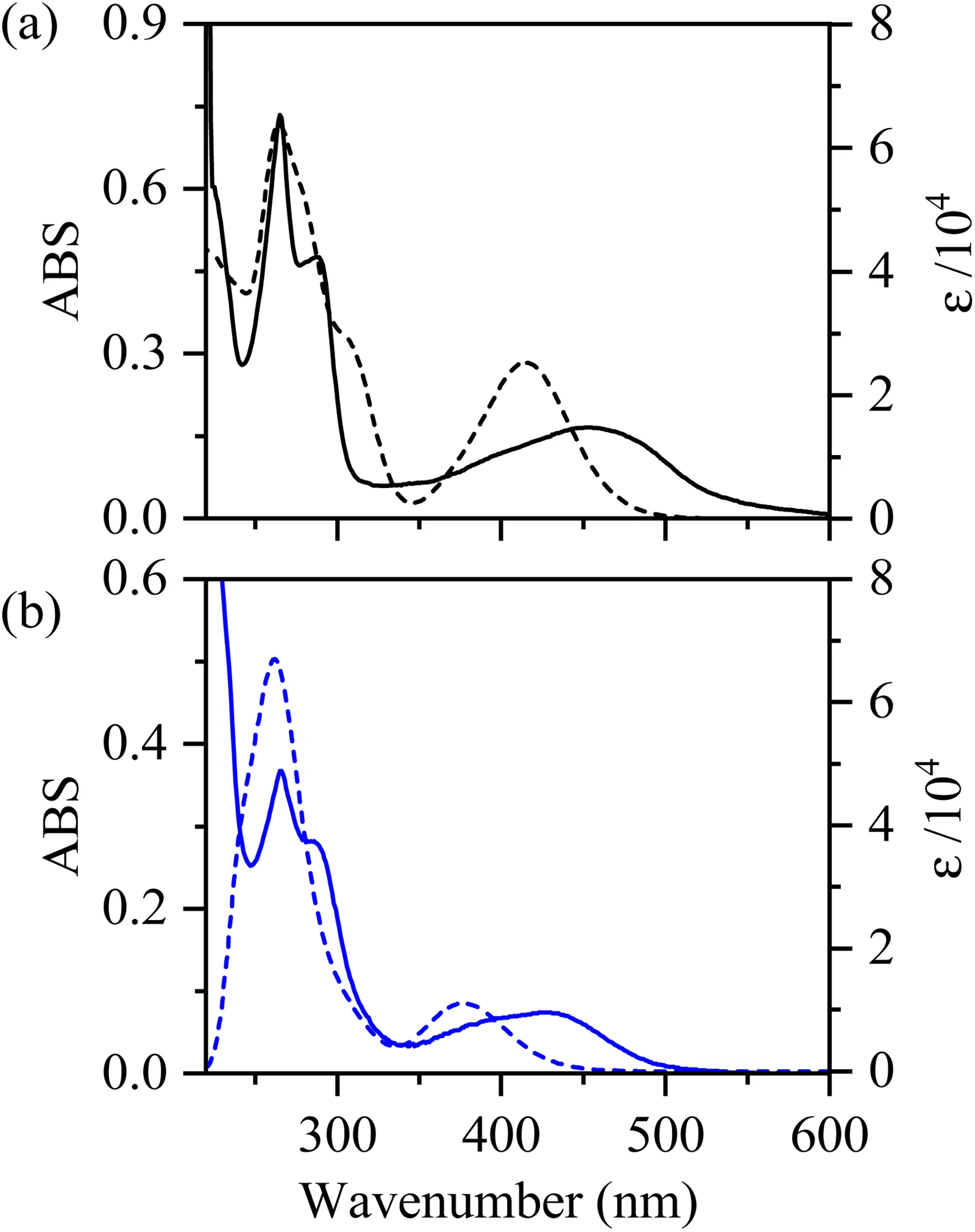
Experimental (solid lines) and calculated (dashed lines) UV-Vis spectra of NAT41 (a) and NAT60 (b) in methanol.

The experimental and calculated UV-Vis spectra of complexes NAT41 and NAT60 in methanol are presented in Fig. 2. As observed, the computed data are in good agreement with the experimental results. Consequently, the band assignments were determined through TD-DFT calculations. The nature of the electronic transitions was determined by analysis of natural transition orbitals (NTO), which provide a compacted representation of the donor–acceptor pair for excitations, as shown in Tables S1 and S2.

Both complexes, as shown in Fig. 2(a) and (b), exhibit strong absorption in the UV region, with maximum absorptions (*λ*_max_) between 220 and 300 nm. According to NTO analysis, in NAT 41, this band is associated with intraligand π–π* transitions involving the 4-Clpy, bpy, and phen ligands (Table S2). In contrast, for NAT60, the band originates from ligand-to-ligand charge transfer (LLCT) transitions from π(phen) to π*(bpy) and from π*(PPH_3_) to π(phen), as well as from metal-to-ligand charge transfer (MLCT) from dπ(Ru) to π*(phen) transitions (Table S3).

In the visible region, both complexes exhibit a broad band associated with MLCT transitions from dπ(Ru) to π*(bpy) and to π*(phen, 4-Clpy) for NAT41 and to π*(phen) for NAT60. The MLCT transitions in NAT60 occur at higher energy than those for NAT41, supporting the conclusion of greater stability, as evidenced by the electrochemical measurements and TD-DFT results. Furthermore, these results are consistent with previously reported data for similar complexes.^40,41^

### 3.2. Photocatalytic activity

The photocatalytic experiments were conducted in a photoreactor equipped with three mercury vapor lamps, each with a nominal power of 20 W, resulting in a total power of 60 W. The lamps were arranged equidistantly, with approximately 13 cm between the irradiation sources, ensuring uniform exposure of the reaction medium to the radiation throughout the experiments. Irradiation was maintained constant over the entire reaction period. The reaction system was connected to a continuous stirring pump with controlled circulation. For each experiment, 200 mL of an aqueous solution containing 25 ppm of Remazol Red Ultra RGB dye were used, to which 100 mg of the photocatalyst were added. The system was kept under constant stirring for 1 h under lamp irradiation. Before the beginning of the reaction, the solution was stirred for 30 min in the absence of light in order to reach adsorption–desorption equilibrium. After this period, the lamps were turned on to start the reaction. The collected aliquots were filtered using 0.22 mm microporous filters and analyzed by UV-Vis spectrophotometry (DR5000-03, HACH). The degradation rate (*D*%) was calculated by eqn (1), where *C*_0_ represents the initial dye concentration and *C*_*t*_ the concentration at each analyzed time.1
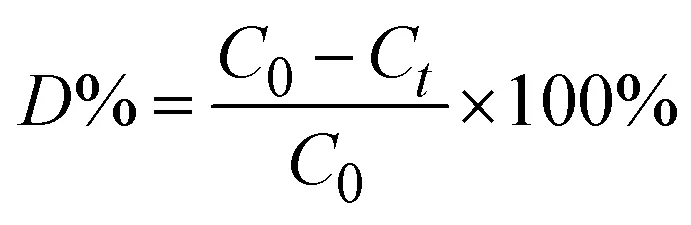


In the photocatalytic test, it was possible to analyze which catalyst showed the best performance in the degradation of the Remazol Red Ultra RGB dye. A decrease in the band with maximum absorption at 514 nm, responsible for the dye coloration, was observed over 1 h of reaction under the action of the catalysts (compound 1 and compound 2). As shown in Fig. 3, the intensity of the absorption band decreased over time in both cases.

**Fig. 3:**
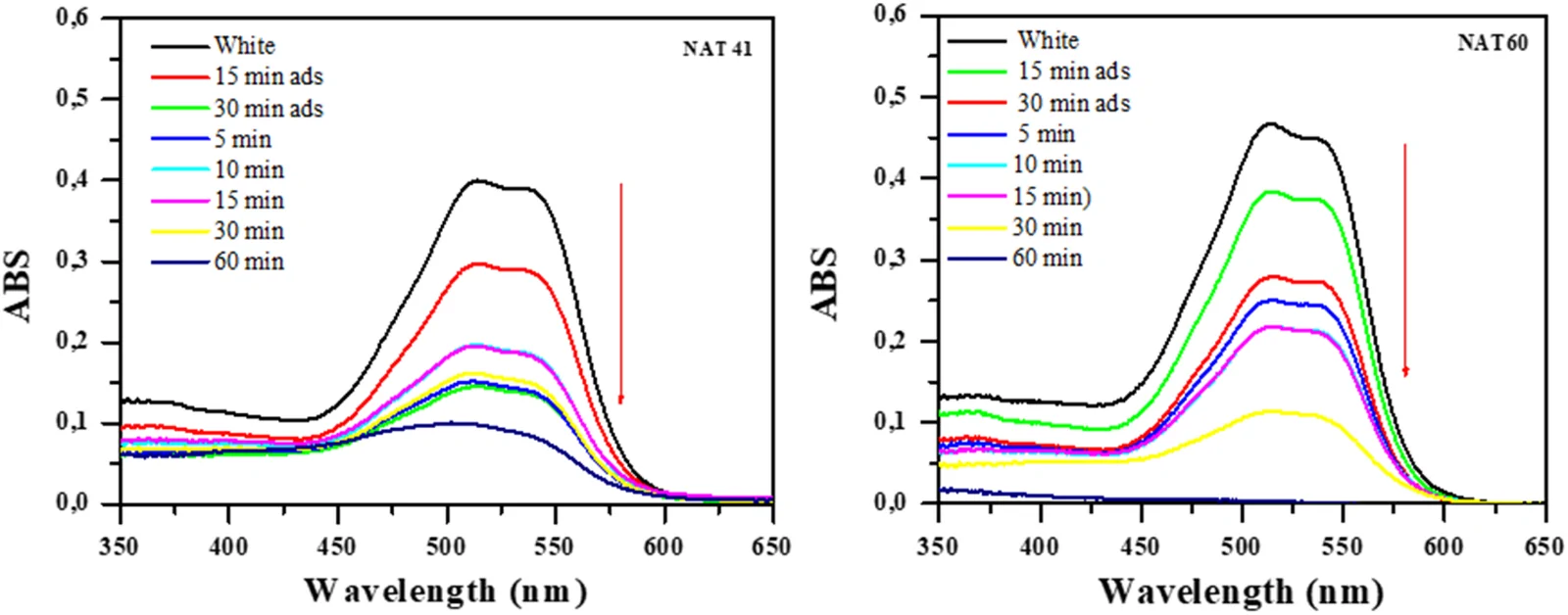
Electronic spectra in the presence of catalysts compound 1 and compound 2 in water, obtained in the 350–650 nm region during the photocatalytic degradation test of the Remazol Red Ultra RGB dye.

Catalyst compound 1 promoted the reduction of the maximum absorption band at 514 nm, with the absorbance decreasing from 0.40 to 0.145 after 30 min of adsorption. After turning on the lamps, a temporary increase in this band was observed, possibly associated with partial dye desorption, followed by a gradual reduction in absorbance, reaching 0.0985 at the end of 60 min of reaction.

Catalyst compound 2 showed the best efficiency, evidenced by the significant reduction of the band at 514 nm throughout the reaction. The initial absorbance of 0.467 decreased to 0.278 after 30 min of adsorption and continued decreasing after the lamps were turned on, reaching 0.00305 at the end of 60 min.

From the analysis of Fig. 4, it can be observed that the presence of the catalysts under light irradiation increases the degradation efficiency of the Remazol Red Ultra RGB dye. Comparing the experiments at the same reaction time, compound 2 showed the best performance, as described in Table 1. Compound 2 reached 100% degradation, while compound 1 showed 75.50% degradation and photolysis presented 16.56% degradation. Fig. 4 shows the comparison of the dye degradation percentages.

**Fig. 4:**
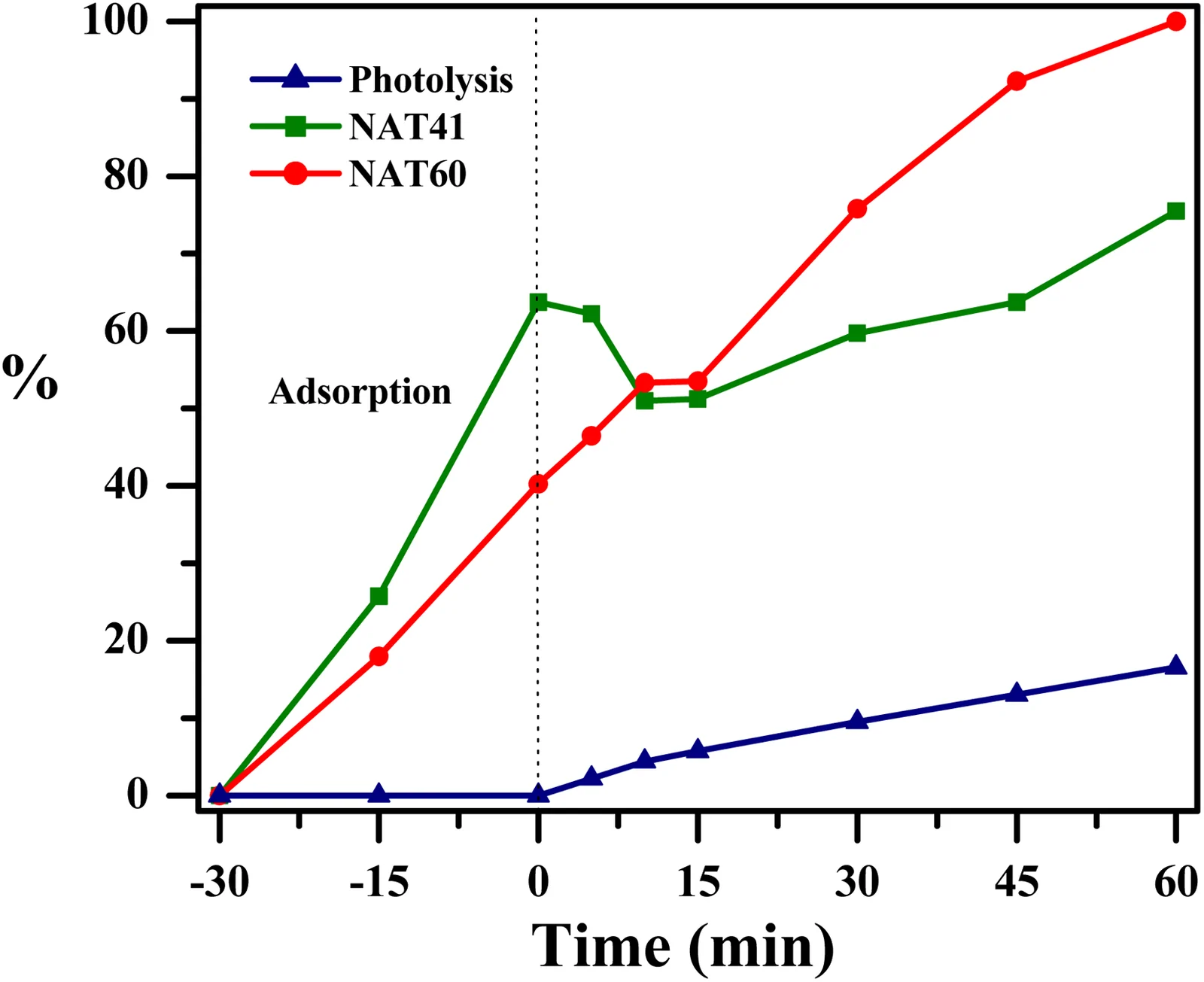
Comparative degradation rate of the catalysts: photolysis without catalyst (blue), compound 1 (green), and compound 2 (red).

**Table 1:** Comparative table of degradation rates for the catalysts: photolysis, compound 1, and compound 2

Photolysis	Compound 1	Compound 2
16.56%	75.50%	100%

In order to enrich the study through association with a mesoporous material, the best-performing catalyst was obtained by wet impregnation onto SBA-15, according to the methodology described by Cavalcanti (2023),^36^ resulting in the material of compound 3. The photocatalytic test was conducted under the same previously established conditions, using 200 mL of aqueous dye solution and 100 mg of catalyst compound 3, with a total reaction time of 1 h and the same sampling intervals.

Fig. 5 shows the electronic spectrum of compound 3 in the range of 350 to 650 nm, evidencing the gradual reduction of the characteristic band at 514 nm. Initially, the absorbance was 0.481, decreasing to 0.225 after 30 min of dye adsorption onto the catalyst. After turning on the lamps, a continuous decrease in absorbance was observed throughout the reaction, reaching the final value of 0.071 at the end of 60 min of irradiation. These results indicate the efficiency of the system in reducing the band at 514 nm over the 1 h reaction period, demonstrating the action of the system in dye degradation.

**Fig. 5:**
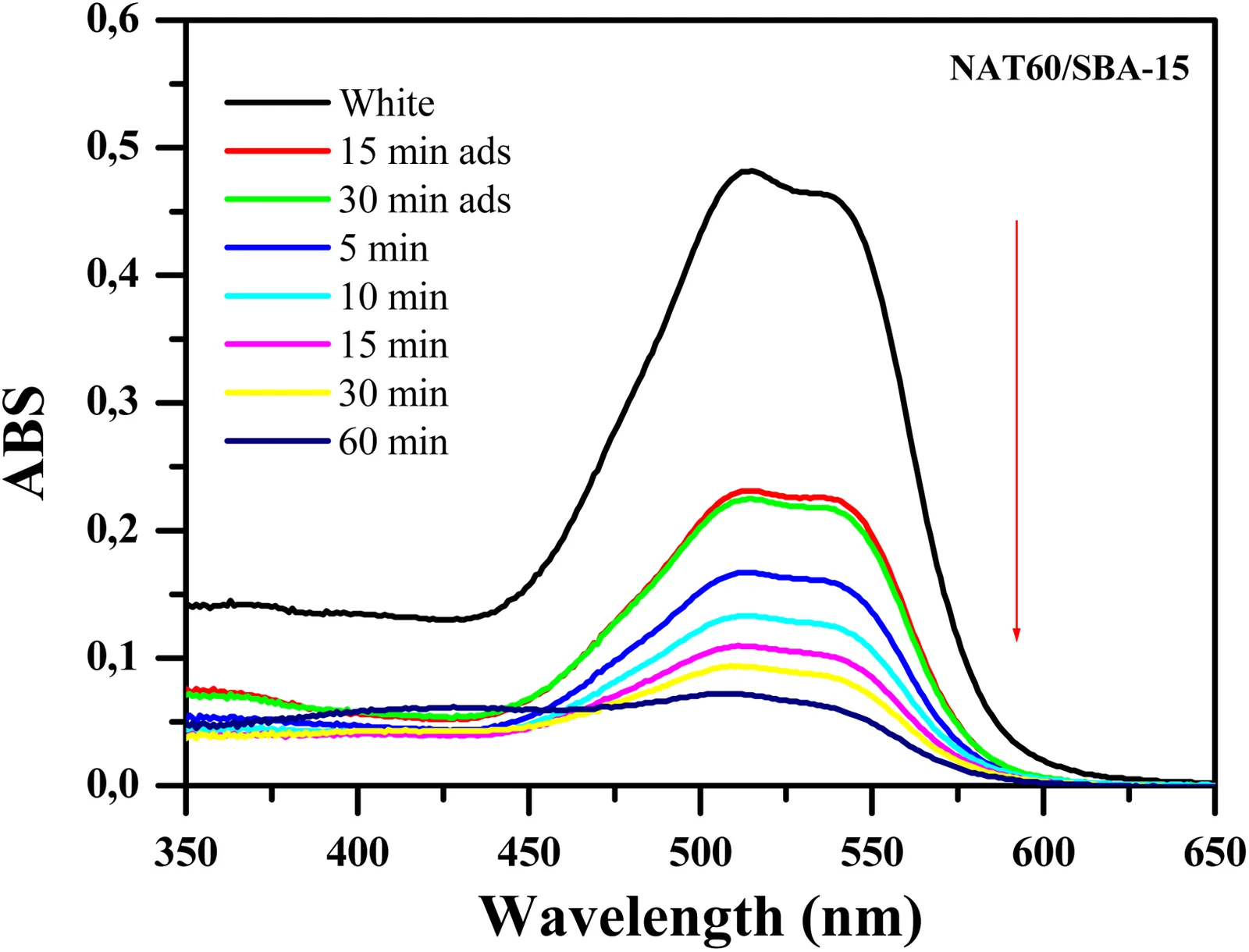
Electronic spectrum in the presence of catalyst compound 3 in water, obtained in the 350–650 nm region during the photocatalytic degradation test of the Remazol Red Ultra RGB dye.

Therefore, Fig. 6 confirms that catalyst compound 3 was efficient in the degradation process, achieving a degradation rate of 85.24% after 60 min of reaction, with no formation of new contaminants.

**Fig. 6:**
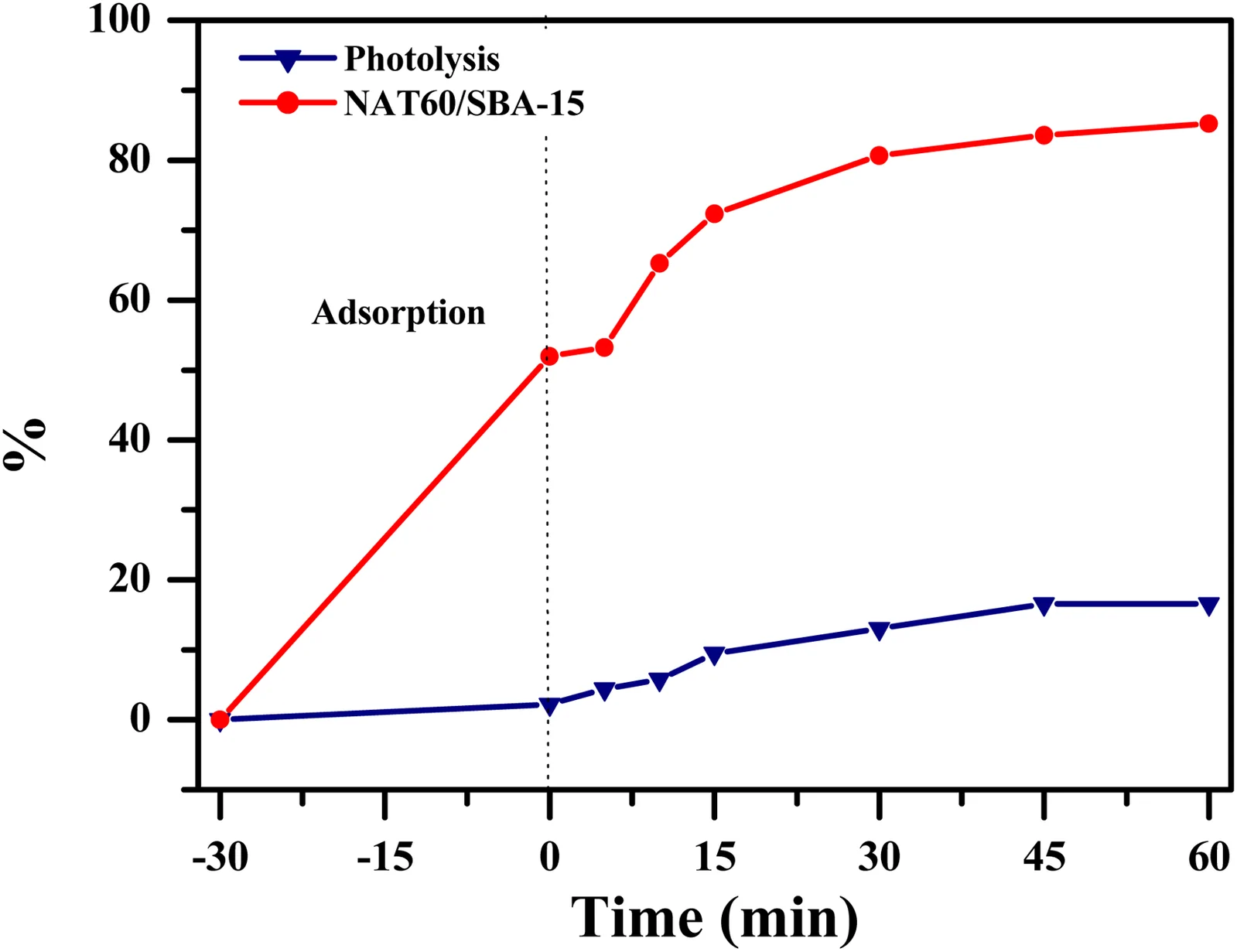
Comparative degradation rate of photolysis (blue) and compound 3 (red).

The kinetic analysis of the dye degradation was performed using zero-order and second-order kinetic models. The fits obtained for the evaluated systems are presented in Fig. 7, while the corresponding kinetic parameters are summarized in Table 2. The NAT60 catalyst showed the best fit to the zero-order model, with an *R*^2^ value of 0.99, whereas the NAT60/SBA-15 system exhibited the best fit to the second-order model, with an *R*^2^ value of 0.97. The rate constants (*k*′) and half-lives (*t*_1/2_) obtained from the kinetic fits are presented in Table 2.

**Fig. 7:**
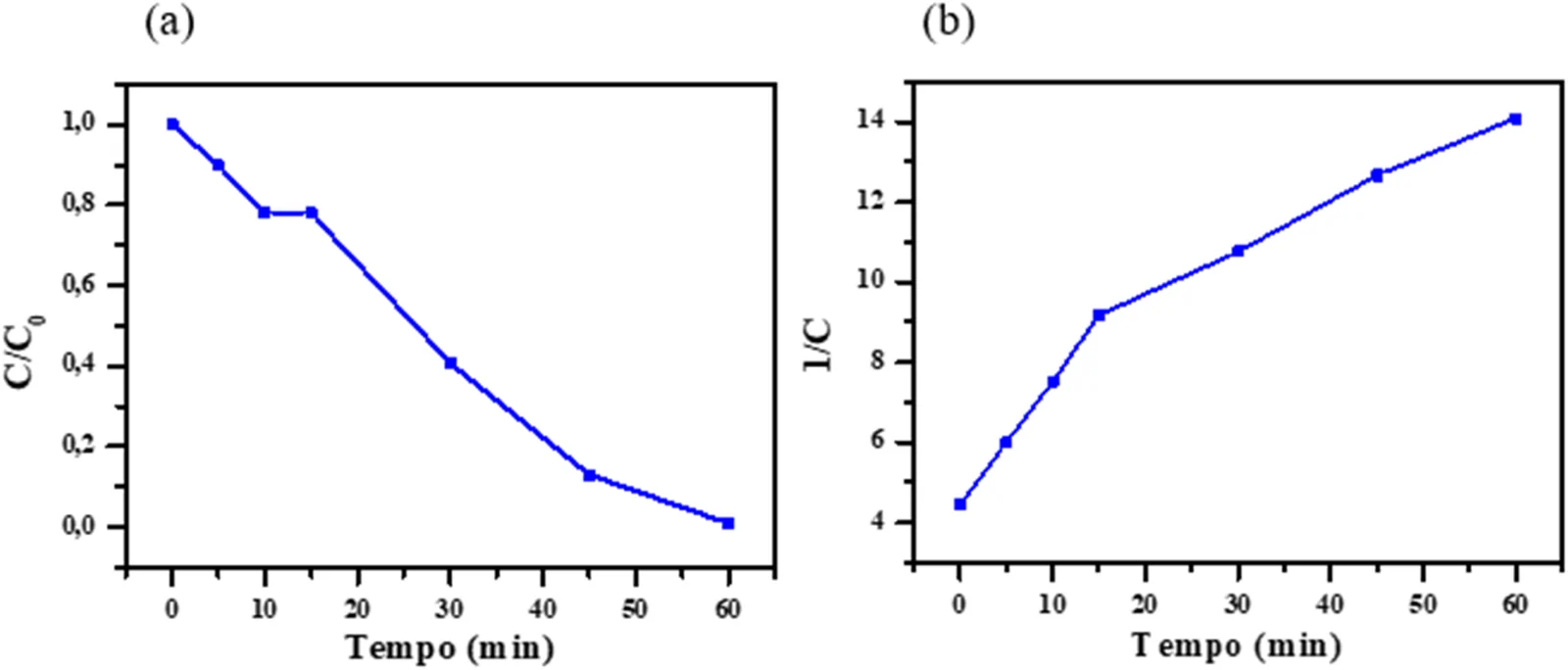
Zero- and second-order kinetic models for dye degradation, respectively: (a) NAT60 and (b) NAT60/SBA-15.

**Table 2:** Kinetic parameters for the dye degradation reaction

Materials	*R* ^2^	*k*′ (min^−1^)	*t*_1/2_ (min)
NAT60	0.99	0.98	0.14
NAT60/SBA-15	0.97	5.62	0.79

Table 3 presents a comparison of different photocatalytic systems employed for the degradation of the Remazol Red Ultra RGB dye, considering degradation efficiency, treatment time, kinetic constant, irradiation source, and operating conditions. The results indicate that the degradation performance depends on both the nature of the photocatalyst and the conditions employed in the process, with degradation efficiencies ranging from 85 to 100% among the systems evaluated. The NAT60 and NAT60/SBA-15 photocatalysts achieved degradation efficiencies of 100% and 85%, respectively, after 60 min of irradiation. Although some systems reported in the literature require shorter treatment times, the materials evaluated in this study exhibit comparable performance. Furthermore, the use of magnetic materials is noteworthy, as it enables their recovery from the reaction medium, representing an additional advantage for applications in wastewater treatment processes.

**Table 3:** Comparison of the photocatalytic activity of different heterogeneous photocatalysts for the degradation of Remazol Red dyes

Photocatalyst	Contaminant	Efficiency (%)	Time (min)	*K* (min^−1^)	Operating conditions	References
NAT60	Remazol red	100	60	0.98	Catalyst loading = 100 mg in 200 mL; dye concentration = 25 mg L^−1^; reaction time = 60 min	This work
NAT60/SBA-15	Remazol red	85	60	5.62	Catalyst loading = 100 mg in 200 mL; dye concentration = 25 mg L^−1^; reaction time = 60 min	This work
SrFe_12_O_19_/SiO_2_-650 (carbon)	Remazol red	100	15	—	Catalyst loading = 40 mg in 200 mL; [H_2_O_2_]= 1 mM; dye concentration= 25 mg L^−1^; initial solution pH = 4; reaction time = 15 min	42
TiO_2_/expanded perlite	Remazol red	100	120	0.0081	Catalyst loading = 100 mg; dye concentration = 10 mg L^−1^; initial solution pH = 5; reaction time = 120 min	43
Sample R-600	Remazol red	100	60	0.042	Catalyst loading = 40 mg in 200 mL; [H_2_O_2_] = 1 mM; dye concentration = 25 mg L^−1^; initial solution pH = 4; reaction time = 60 min	44
CoFe_2_O_4_/Fe_2_O_3_-KIT-6 – with H_2_O_2_	Remazol red	98	120	0.066	Catalyst loading = 0.5 g L^−1^; [H_2_O_2_] = 20 mM; dye concentration = 25 mg L^−1^; initial solution pH = 3; reaction time = 120 min	45

The SBA-15 support exhibited the characteristic rod-like morphology commonly reported for this material. After impregnation with NAT60, some morphological modifications, particularly increased surface roughness, were observed. Nevertheless, the fibrous/rod-like morphology was largely preserved, indicating that the impregnation procedure did not result in severe morphological changes. This preservation may be associated with the absence of a calcination step after impregnation. The SEM images of the pristine SBA-15 and NAT60/SBA-15 materials are presented in Fig. S3 of the SI.

## 4. Conclusion

According to the objectives of this work, it was observed that the developed ruthenium-based complexes were able to act as photocatalysts in the degradation of the Remazol Red Ultra RGB dye. The photocatalytic assays demonstrated the efficiency of the synthesized materials, with emphasis on catalyst compound 2, which showed the greatest reduction in the absorption band at 514 nm, indicating high photocatalytic activity compared to the other evaluated systems.

The experimental results obtained by UV-Vis spectroscopy, infrared spectroscopy, and cyclic voltammetry were corroborated by computational studies based on DFT and TD-DFT, which provided a consistent interpretation of the electronic transitions, vibrational modes, and electrochemical behavior of the complexes. This agreement between theoretical and experimental data contributed to a deeper understanding of the structural, electronic, and photochemical properties of the developed materials.

In order to explore the advantages of a mesoporous matrix, especially the higher surface area and better dispersion of catalytic sites, favoring the contact between the catalyst and dye molecules, the impregnation of the best-performing catalyst into a mesoporous material, through the preparation of compound 3 material, contributed to the enrichment of the study, allowing the evaluation of the influence of this support on photocatalytic performance. The results demonstrated that the compound 3 system maintained high efficiency in dye degradation, highlighting the potential of the association between metal complexes and mesoporous supports for applications in effluent treatment.

In general, the obtained results confirm the potential of ruthenium complexes and their materials as promising photocatalysts for dye degradation in aqueous systems, contributing to the development of sustainable strategies for industrial effluent treatment.

## Conflicts of interest

There are no conflicts to declare.

## Supplementary Material

RA-OLF-D6RA06712C-s001

## Data Availability

The data supporting this article have been included as part of the supplementary information (SI). Supplementary information: additional spectroscopic characterization data, infrared spectra, and experimental and calculated electronic transition data supporting the conclusions of this work. See DOI: https://doi.org/10.1039/d6ra06712c.
