# Supplementary material for: Development of new ruthenium complex-based photocatalysts for the degradation of Remazol Red Ultra RGB
Source: RSC Adv. 2026 Oct 28;16(49):66972–80. doi: 10.1039/d6ra06712c (PMC13715866; doi:10.1039/d6ra06712c)
Supplement: Supplementary file 1 [file RA-016-D6RA06712C-s001.pdf]

Supplementary Information (SI) for RSC Advances.
This journal is © The Royal Society of Chemistry

Electronic Supplementary Information (ESI)

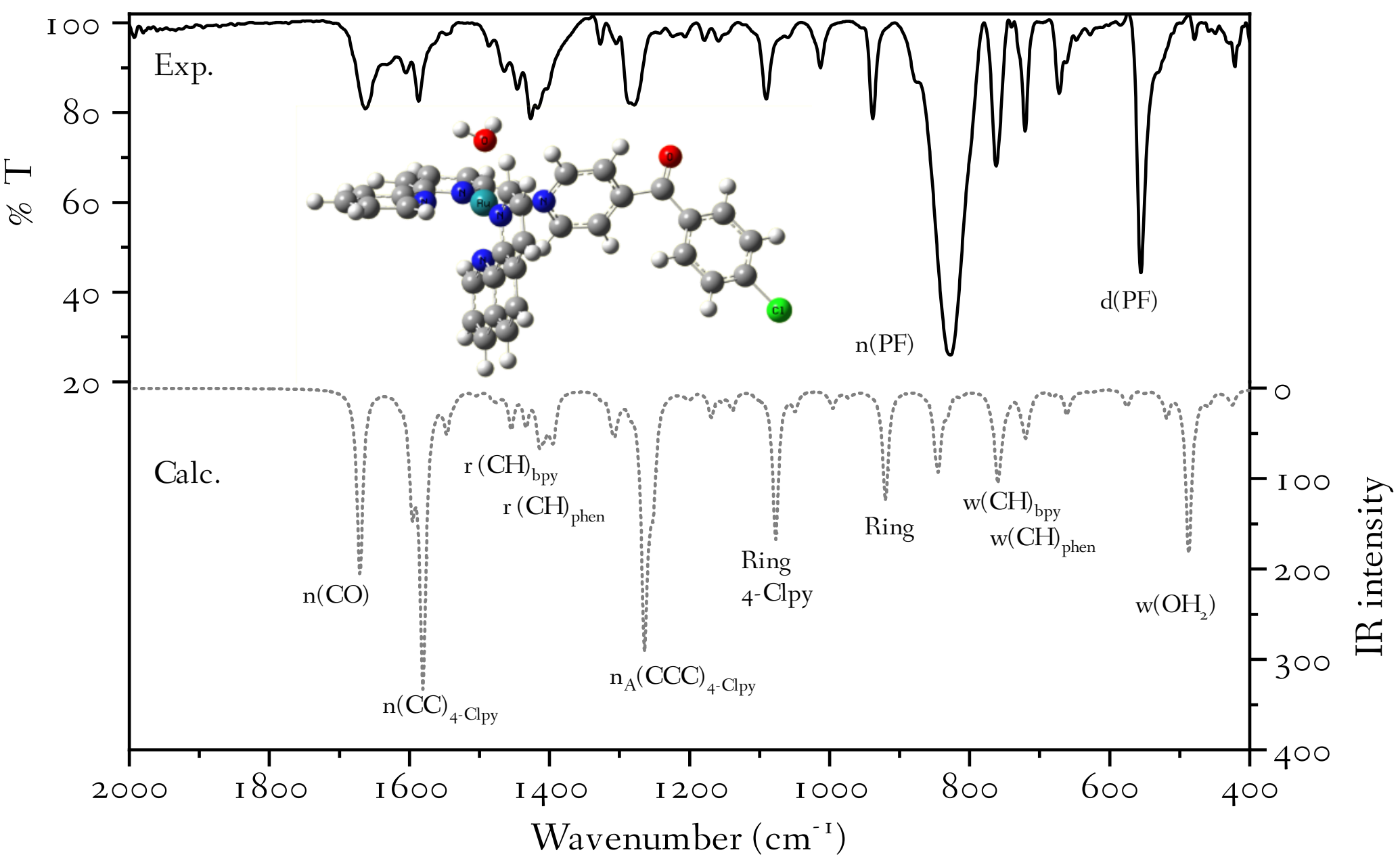

Figure S1. Experimental and calculated infrared spectra of NAT41 and optimized structure of the complex.

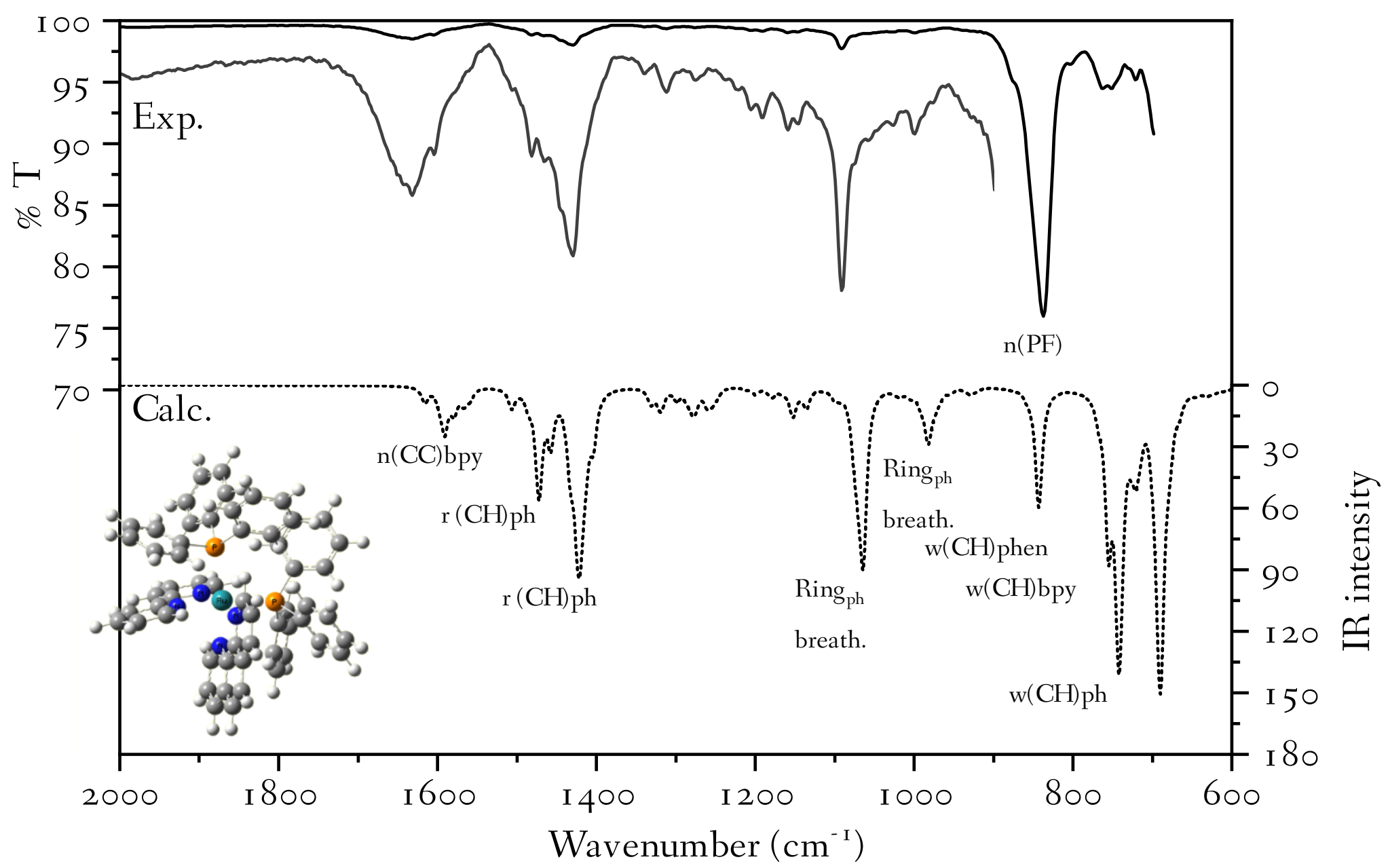

Figure S2. Experimental and calculated infrared spectra of NAT41 and NAT60 complexes. Insets: optimized structures of the complexes.

Table S1. Calculated wavenumbers values of selected IR bands and their respective assignments.

NAT41		NAT60		assignment
Calc.	Exp.	Calc.	Exp.	
1670	1662			ν(CO)
		1590	1631	ν(CC)$_{\text{bpy}}$
1579	1586			ν(CC)$_{\text{4-pyCl}}$
		1471	1481	ρ(CH)$_{\text{ph}}$
		1419	1429	ρ(CH)$_{\text{ph}}$
1413	1427			ρ(CH)$_{\text{phen}}$
1393	1402$_{\text{sh}}$			ρ(CH)$_{\text{bpy}}$
1306	1340			ν(CC)$_{\text{4-pyCl}}$
1263	1280			ν$_{\text{a}}$(CCC)$_{\text{4-pyCl}}$
1076	1089			(ring breath.)$_{\text{4-pyCl}}$
942	935			(ring breath.)$_{\text{4-pyCl}}$
		1069	1091	(ring breath.)$_{\text{ph}}$
		982	999	(ring breath.)$_{\text{ph}}$
843	878	842	873$_{\text{sh}}$	ω(CH)$_{\text{phen}}$
-	826	-	837	ν$_{\text{a}}$(PF)
759	c	753	761	ω(CH)$_{\text{bpy}}$
		741	750	ω(CH)$_{\text{ph}}$
717	718			ω(CH)$_{\text{phen}}$
		689	-	ω(CH)$_{\text{ph}}$
-	555	-	-	δ(PF)
		508	-	τ(CH)$_{\text{ph}}$
485	480			ω(OH₂)

ν = stretching, ω = wagging, τ = twisting, δ = scissoring, ρ = rocking modes, $_{\text{a}}$ = asymmetric, and sh = shoulder

Table S2 - Experimental and calculated singlet excited-state transitions data for NAT41.

Complex	Wavenumber (nm)		Major MO contrib	NTO		Assignment
	Exp.	Calc. (*f*)	(%)	donor	acceptor	
NAT41	477	456 (0.0089)	H→L+1 (65%)	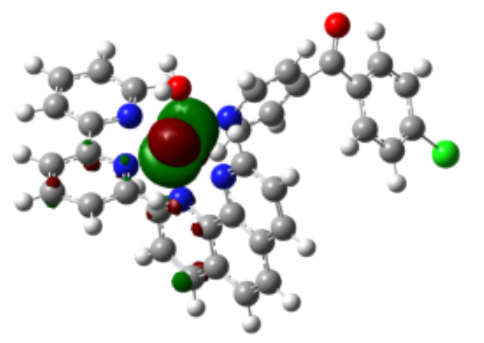	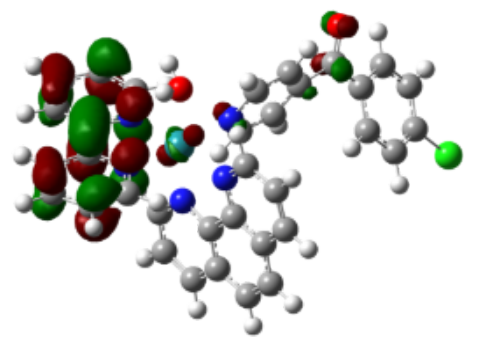	MLCT (bpy)
	449	420 (0.1051)	H-1→L (48%)	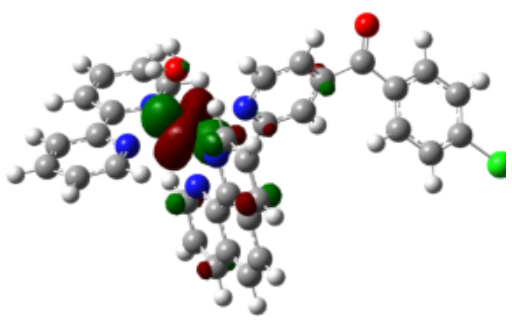	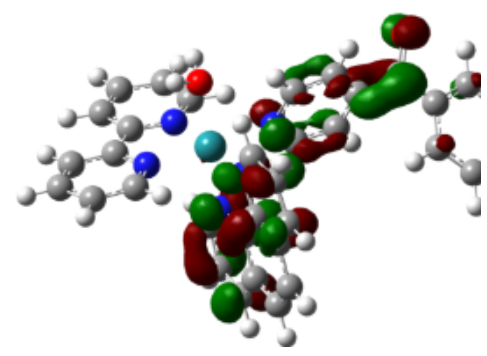	MLCT (phen,4-pyCl)
	395	412 (0.1125)	H-2→L+1 (40%)	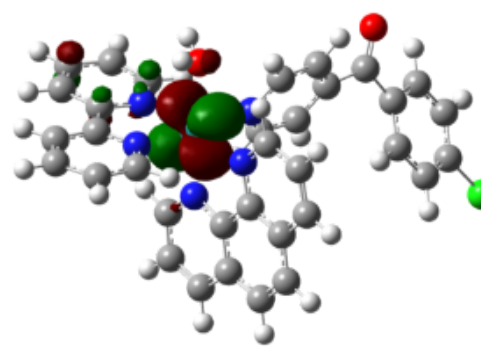	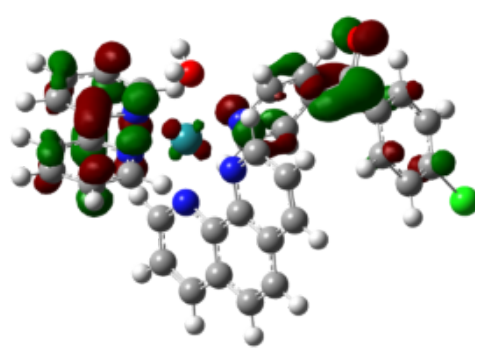	MLCT (bpy,4-pyCl)
	290	309 (0.1496)	H-3→L (58%)	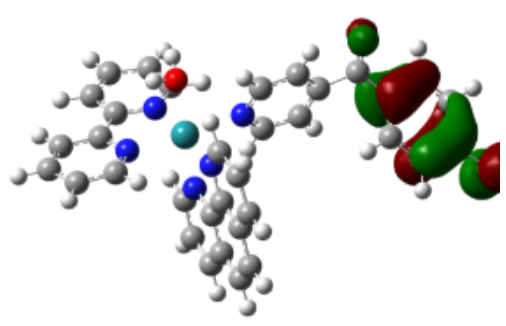	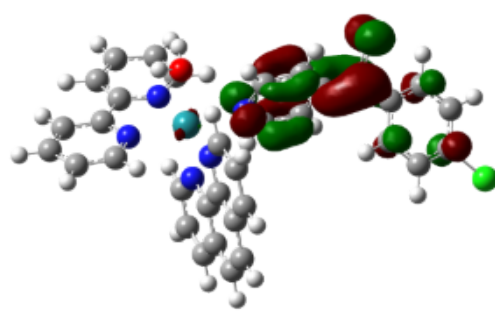	ICT(4-pyCl)
		280 (0.341)	H-5→L+1 (28%) H-2→L+7 (29%)	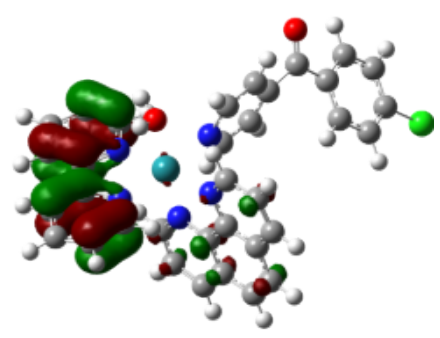	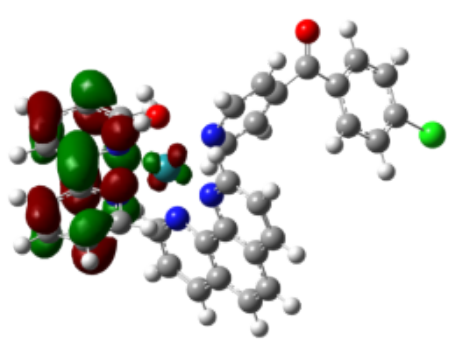	IL(bpy)
	266	261 (0.6631)	H-6→L+2 (41%)	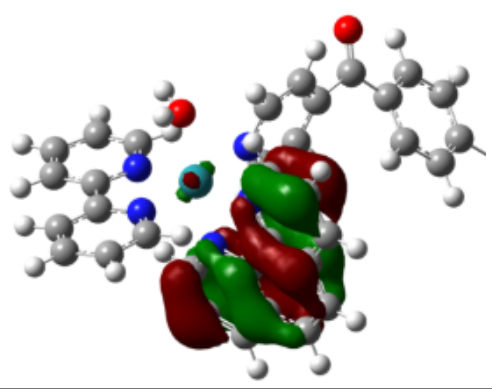	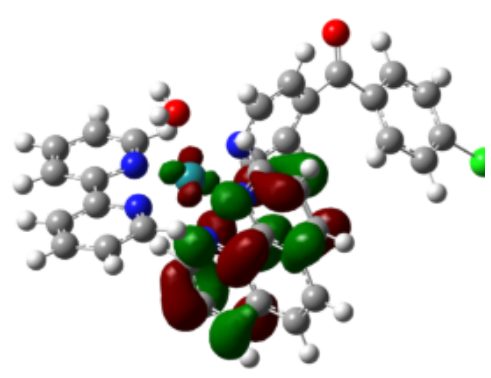	IL(phen)

Table S3 - Experimental and calculated singlet excited-state transitions data for NAT60.

Complex	Wavenumber (nm)	Major MO contrib.	NTO	Assignment

	Exp.	Calc. (*f*)	(%)	donor	acceptor	
NAT60	427	390 (0.0417)	H-2→L (37%) H-2→L+1 (28%)	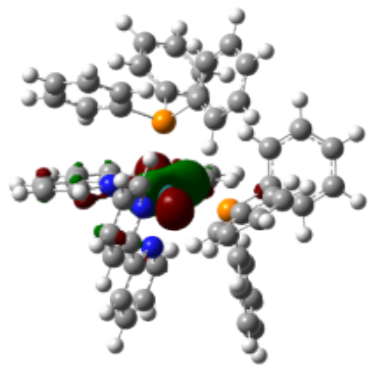	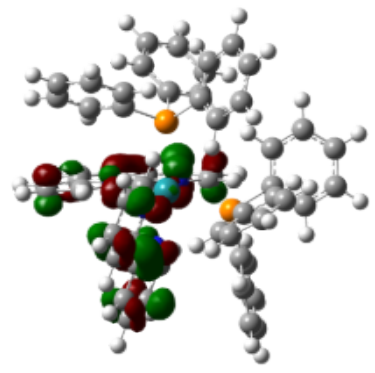	MLCT (phen)
	394	369 (0.0578)	H→L+2 (63%)	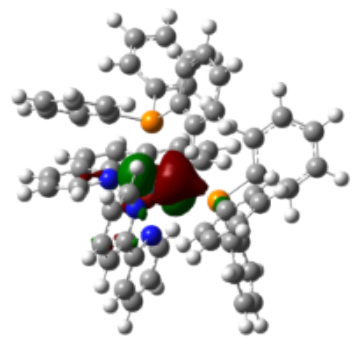	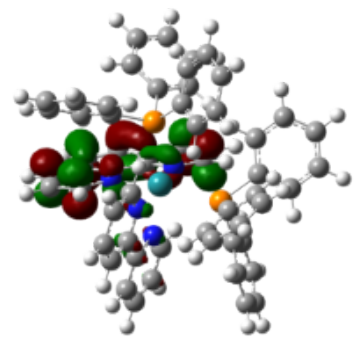	MLCT (phen)
	287	299 (0.0341)	H-2→L+3 (26%) H→L+5 (46%)	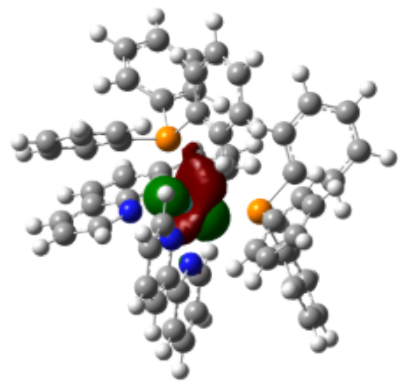	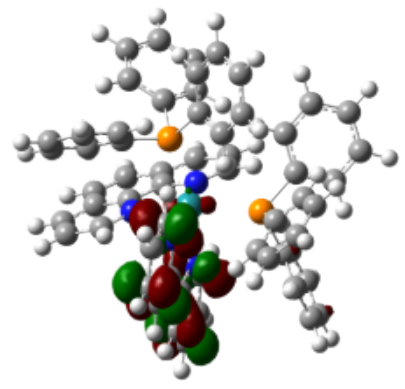	MLCT (phen)
	267	281 (0.0243)	H-17→L (48%)	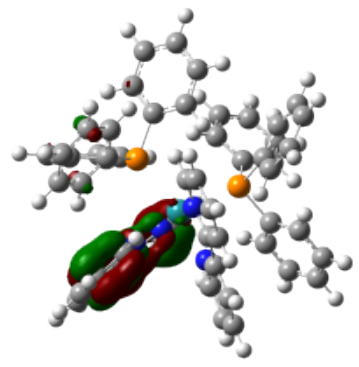	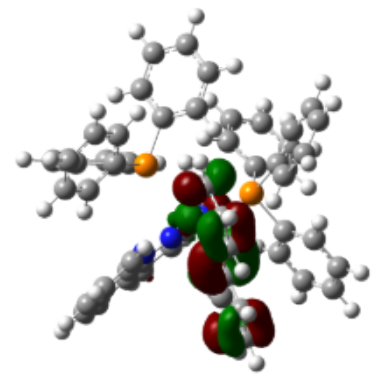	LLCT (phen→bpy)
		262 (0.0988)	H-16→L+2 (24%)	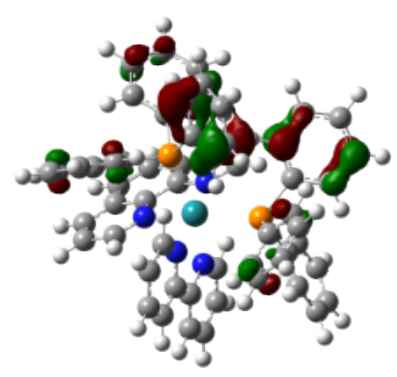	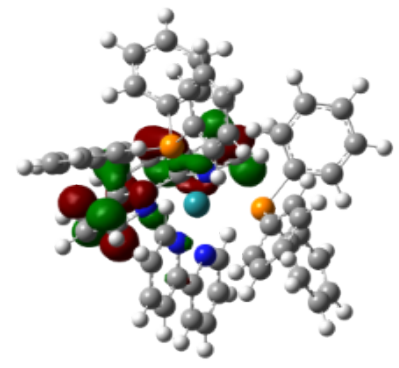	LLCT (PPh→phen)

Figure S3 SEM images of (A) pristine SBA-15 at 1 μm; (B) NAT60/SBA-15 at 1 μm; (C) pristine SBA-15 at 5 μm; and (D) NAT60/SBA-15 at 5 μm.

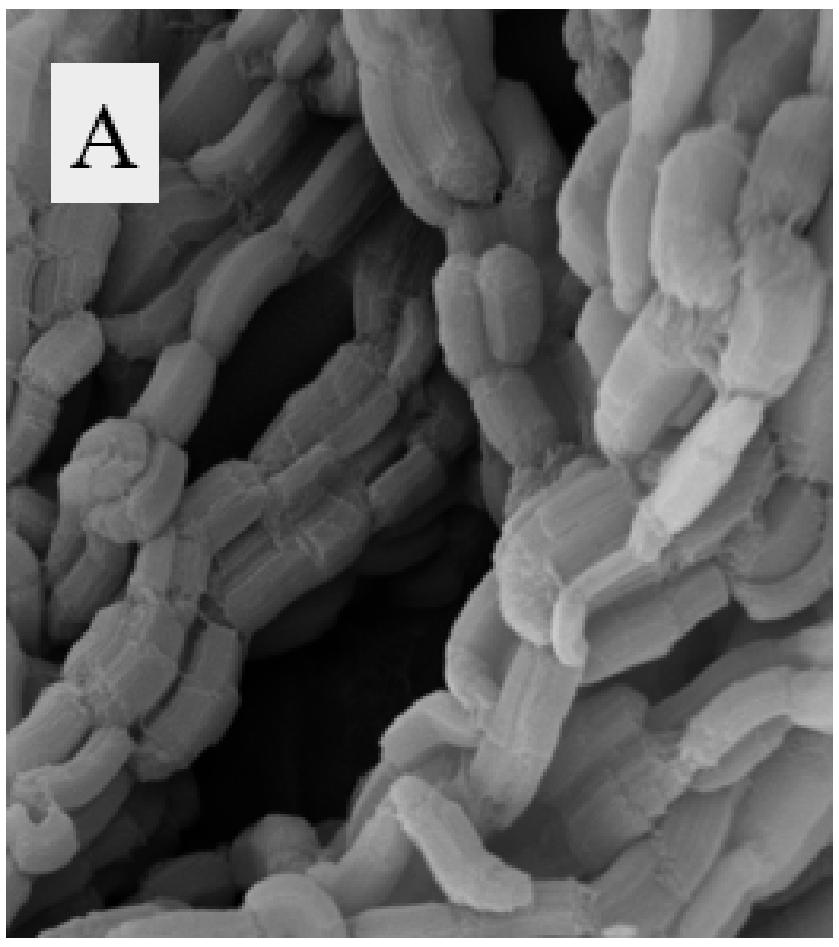

1μm SBA-15 pura

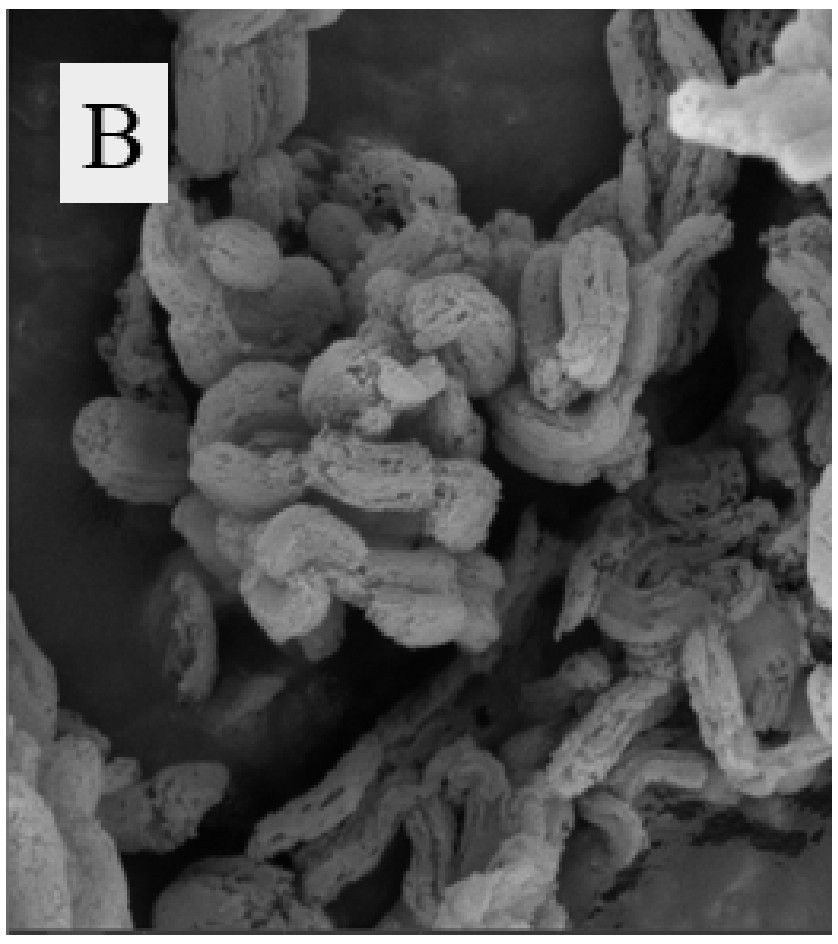

1μm NAT60/SBA-15

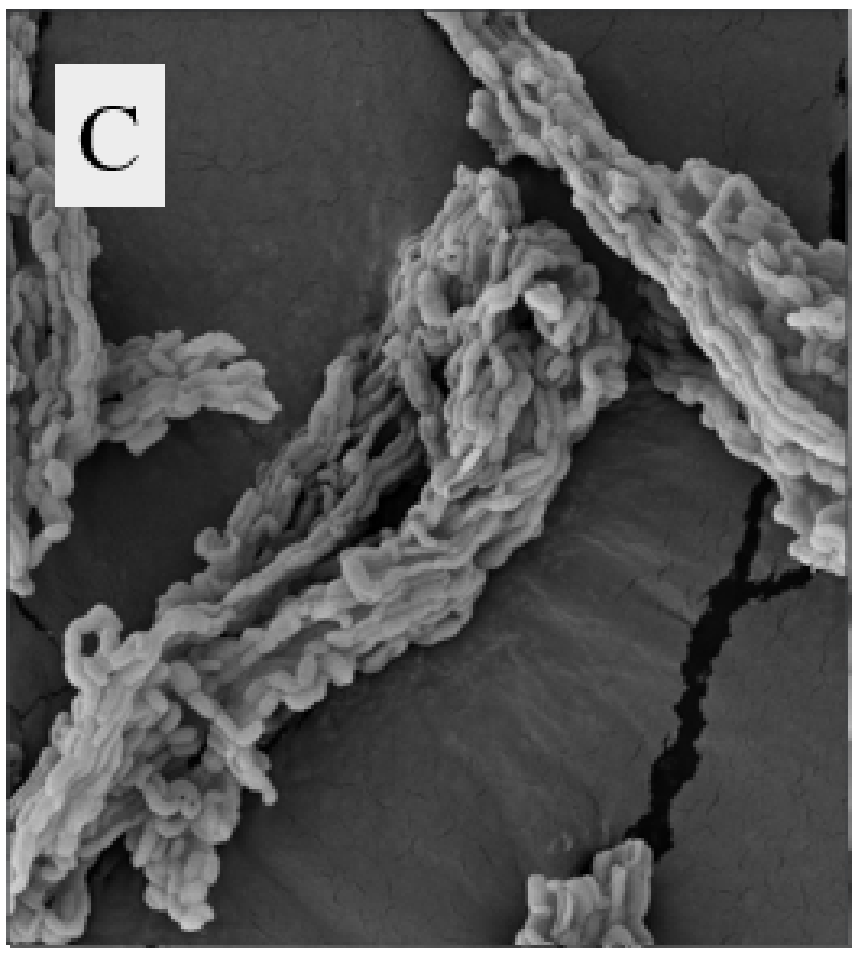

5μm SBA-15 pura

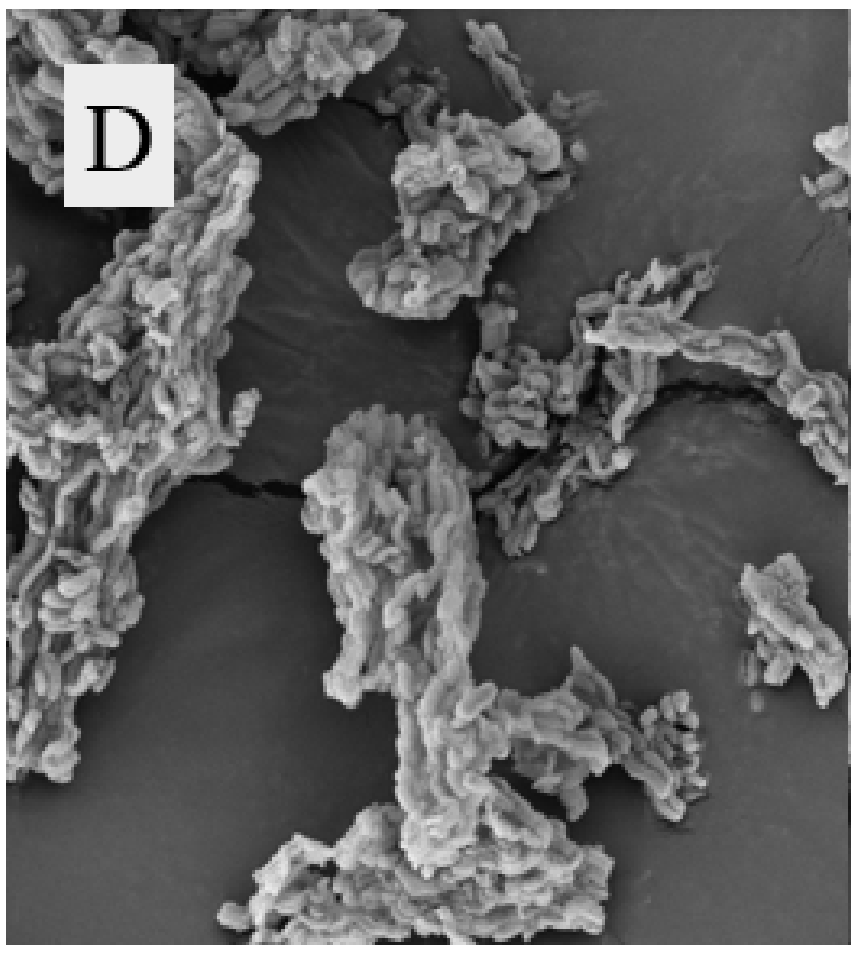

5μm NAT60/SBA-15
