# Supplementary material for: Development of new ruthenium complex-based photocatalysts for the degradation of Remazol Red Ultra RGB
Source: RSC Adv. 2026 Oct 28. Online ahead of print. doi: 10.1039/d6ra06712c (PMC13715866; doi:10.1039/d6ra06712c)
Supplement: Supplementary file 1 [file RA-OLF-D6RA06712C-s001.pdf]

Supplementary Information (SI) for RSC Advances.
This journal is © The Royal Society of Chemistry

Electronic Supplementary Information (ESI)

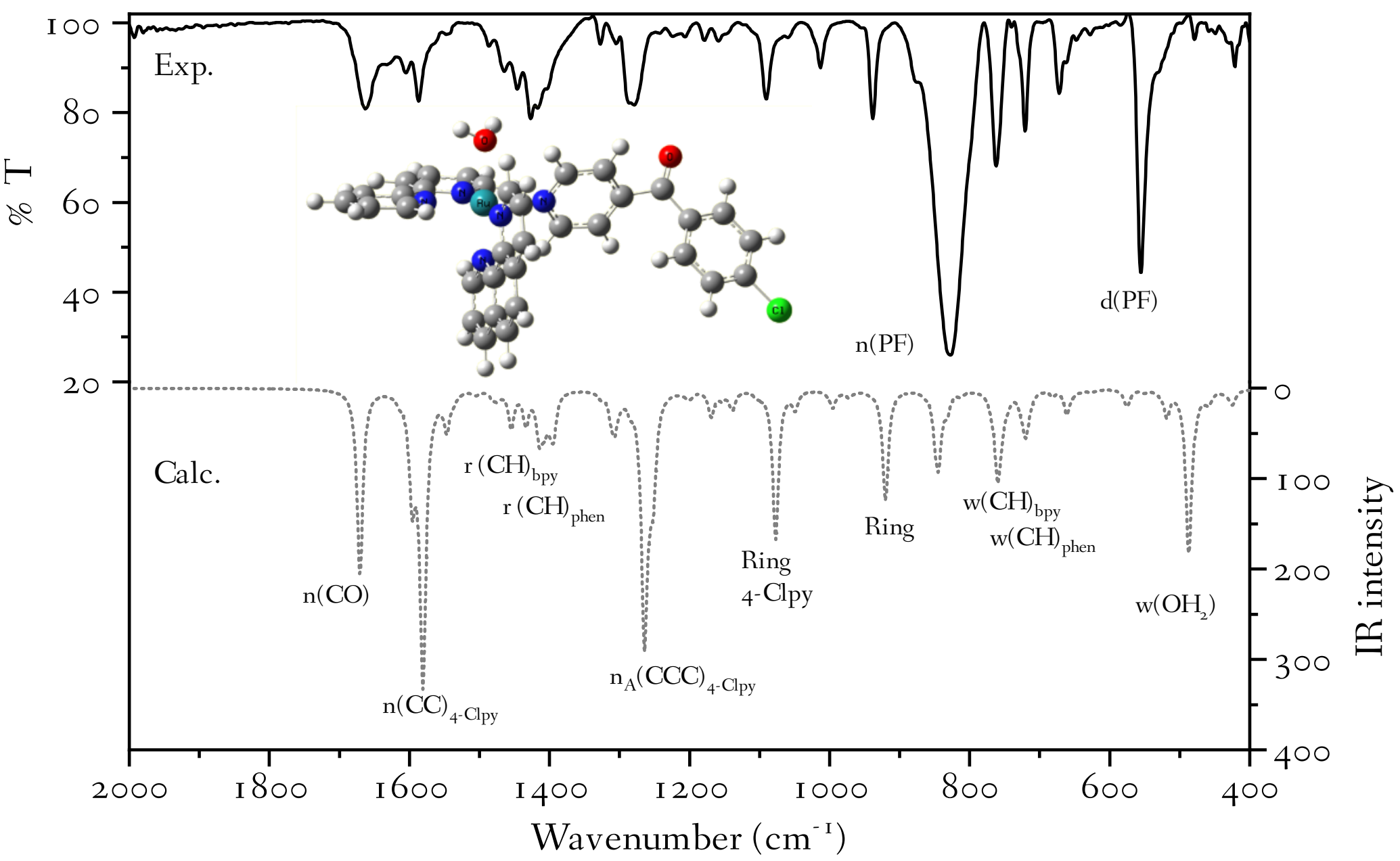


Figure S1. Experimental and calculated infrared spectra of NAT41 and optimized structure of the complex.

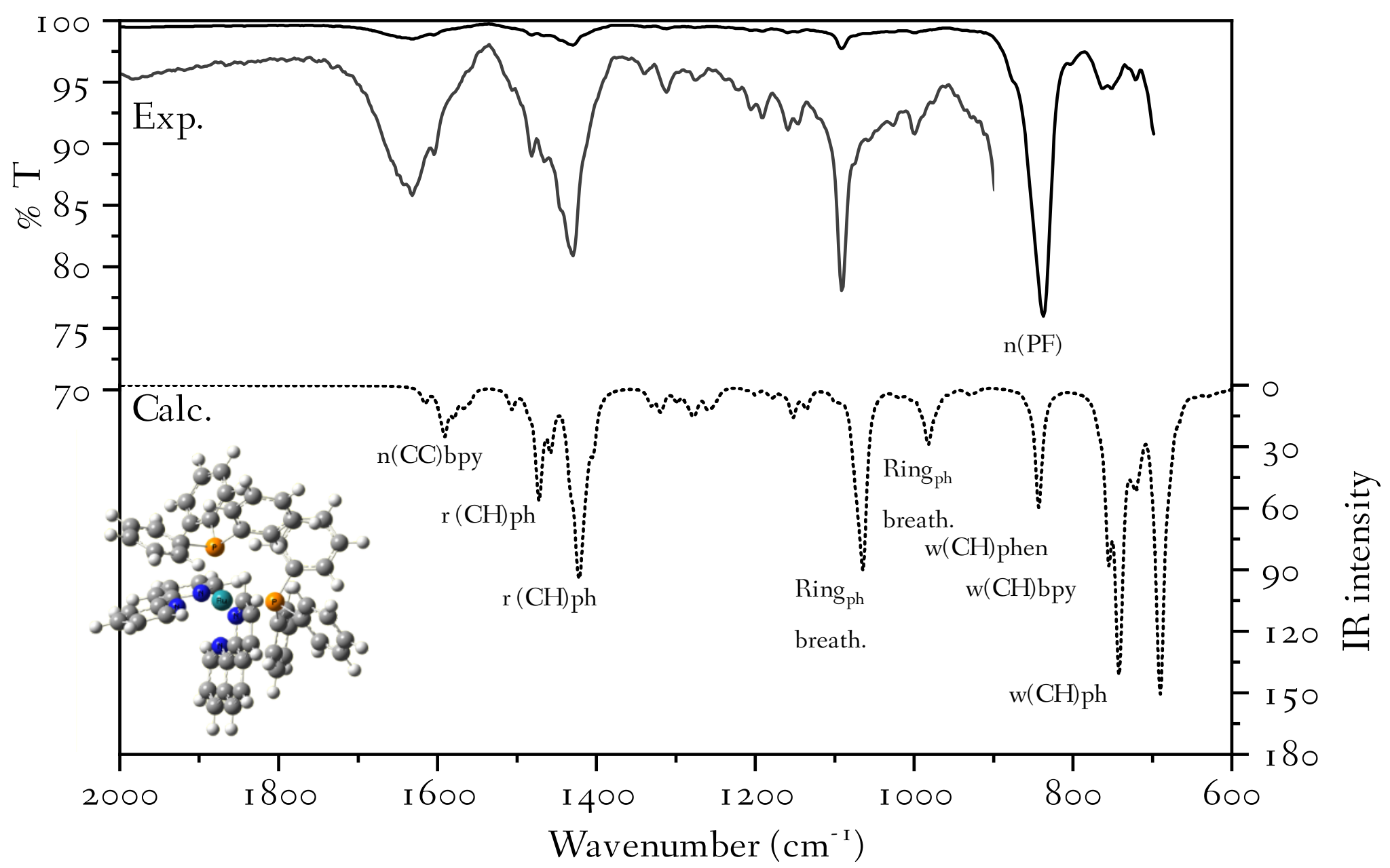

Figure S2. Experimental and calculated infrared spectra of NAT41 and NAT60 complexes. Insets: optimized structures of the complexes.

Table S1. Calculated wavenumbers values of selected IR bands and their respective assignments.

| NAT41 | | NAT60 | | assignment |
|---|---|---|---|---|
| Calc. | Exp. | Calc. | Exp. | |
| 1670 | 1662 | | | ν(CO) |
| | | 1590 | 1631 | ν$(CC)_{bpy}$ |
| 1579 | 1586 | | | ν$(CC)_{4\text{-}pyCl}$ |
| | | 1471 | 1481 | ρ$(CH)_{ph}$ |
| | | 1419 | 1429 | ρ$(CH)_{ph}$ |
| 1413 | 1427 | | | ρ$(CH)_{phen}$ |
| 1393 | $1402_{sh}$ | | | ρ$(CH)_{bpy}$ |
| 1306 | 1340 | | | ν$(CC)_{4\text{-}pyCl}$ |
| 1263 | 1280 | | | ν$_a(CCC)_{4\text{-}pyCl}$ |
| 1076 | 1089 | | | (ring breath.)$_{4\text{-}pyCl}$ |
| 942 | 935 | | | (ring breath.)$_{4\text{-}pyCl}$ |
| | | 1069 | 1091 | (ring breath.)$_{ph}$ |
| | | 982 | 999 | (ring breath.)$_{ph}$ |
| 843 | 878 | 842 | $873_{sh}$ | ω$(CH)_{phen}$ |
| - | 826 | - | 837 | ν$_a$(PF) |
| 759 | c | 753 | 761 | ω$(CH)_{bpy}$ |
| | | 741 | 750 | ω$(CH)_{ph}$ |
| 717 | 718 | | | ω$(CH)_{phen}$ |
| | | 689 | - | ω$(CH)_{ph}$ |
| - | 555 | - | - | δ(PF) |
| | | 508 | - | τ$(CH)_{ph}$ |
| 485 | 480 | | | ω(OH₂) |

$\nu$ = stretching, ω = wagging, τ = twisting, δ = scissoring, ρ = rocking modes, $_a$ = asymmetric, and sh = shoulder

Table S2 - Experimental and calculated singlet excited-state transitions data for NAT41.

| Complex | Wavenumber (nm) | | Major MO contrib | NTO | | Assignment |
|---|---|---|---|---|---|---|
| | Exp. | Calc. (*f*) | (%) | donor | acceptor | |
| NAT41 | 477 | 456 (0.0089) | H→L+1 (65%) | 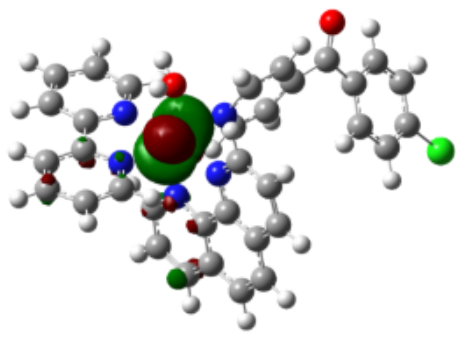 | 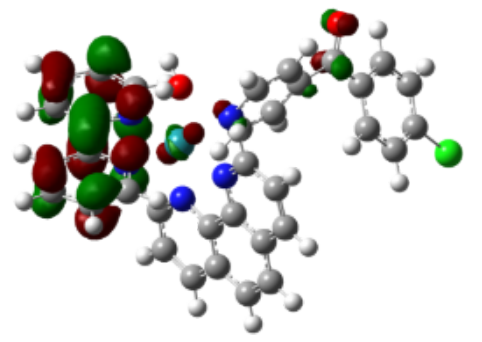 | MLCT (bpy) |
| | 449 | 420 (0.1051) | H-1→L (48%) | 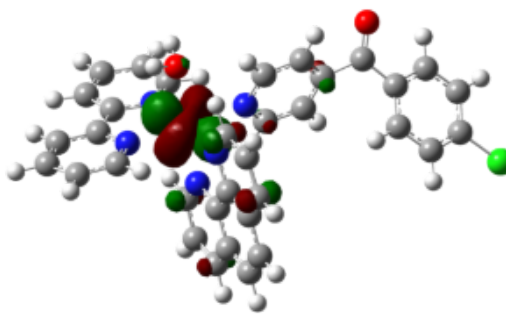 | 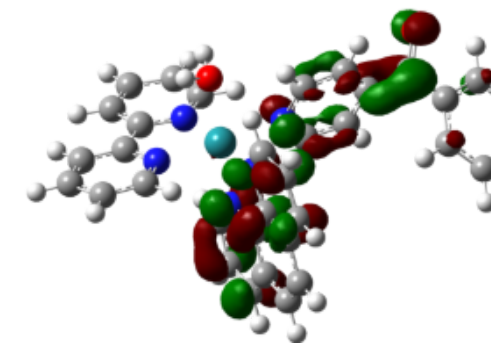 | MLCT (phen,4-pyCl) |
| | 395 | 412 (0.1125) | H-2→L+1 (40%) | 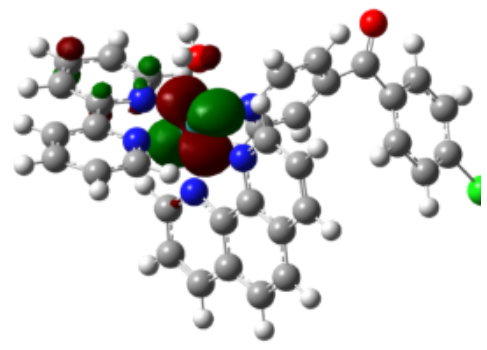 | 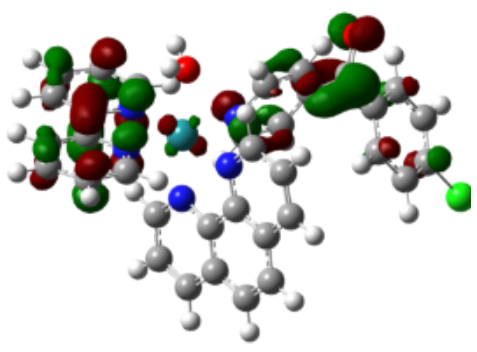 | MLCT (bpy,4-pyCl) |
| | 290 | 309 (0.1496) | H-3→L (58%) | 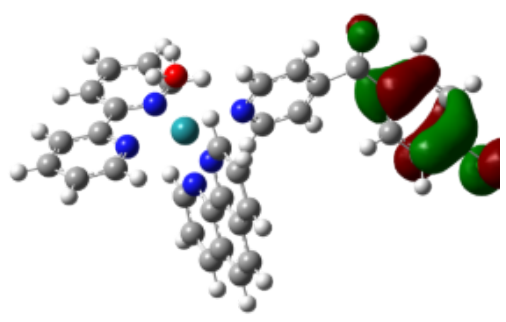 | 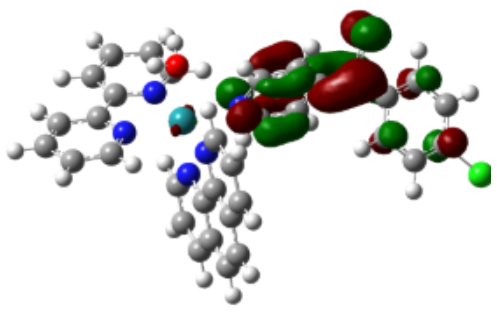 | ICT(4-pyCl) |
| | | 280 (0.341) | H-5→L+1 (28%) H-2→L+7 (29%) | 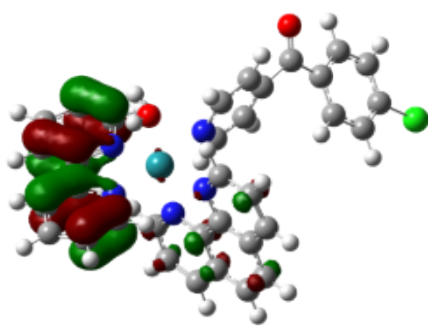 | 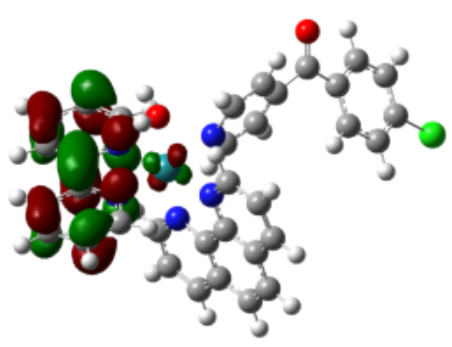 | IL(bpy) |
| | 266 | 261 (0.6631) | H-6→L+2 (41%) | 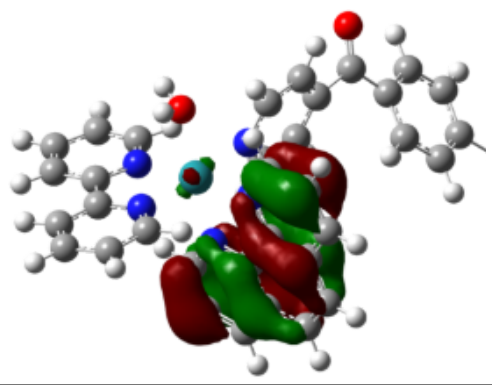 | 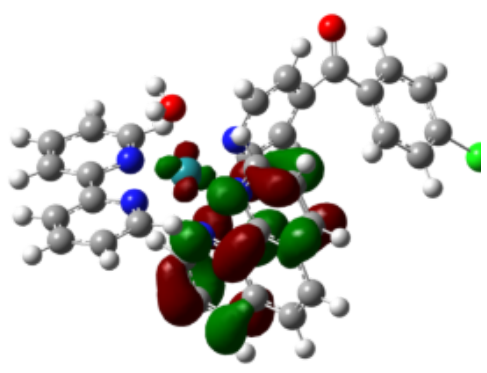 | IL(phen) |

Table S3 - Experimental and calculated singlet excited-state transitions data for NAT60.

| Complex | Wavenumber (nm) | Major MO contrib. | NTO | Assignment |
|---|---|---|---|---|

| | Exp. | Calc. (*f*) | (%) | donor | acceptor | |
|---|---|---|---|---|---|---|
| NAT60 | 427 | 390 (0.0417) | H-2→L (37%) H-2→L+1 (28%) | 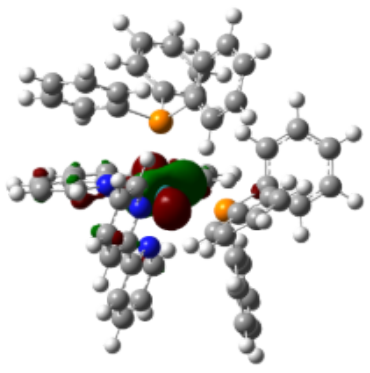 | 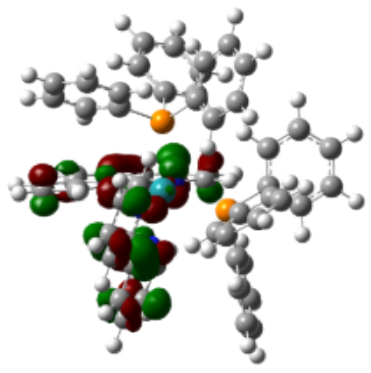 | MLCT (phen) |
| | 394 | 369 (0.0578) | H→L+2 (63%) | 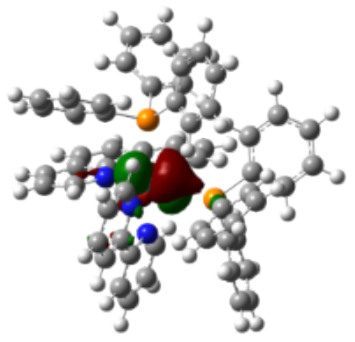 | 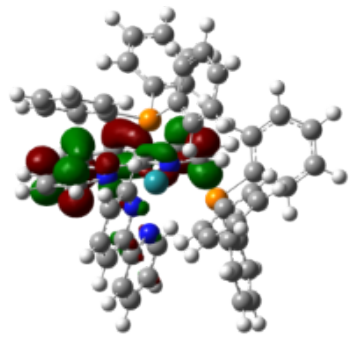 | MLCT (phen) |
| | 287 | 299 (0.0341) | H-2→L+3 (26%) H→L+5 (46%) | 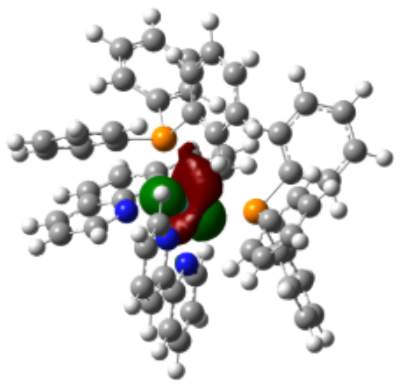 | 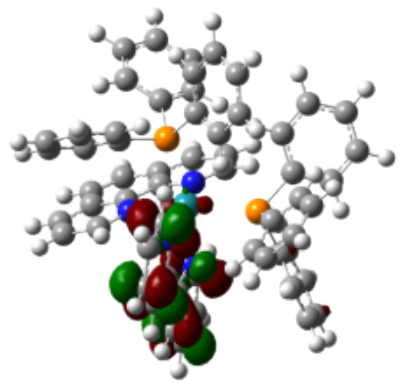 | MLCT (phen) |
| | 267 | 281 (0.0243) | H-17→L (48%) | 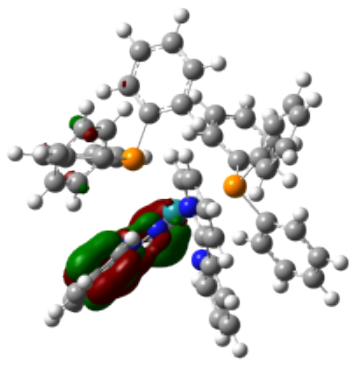 | 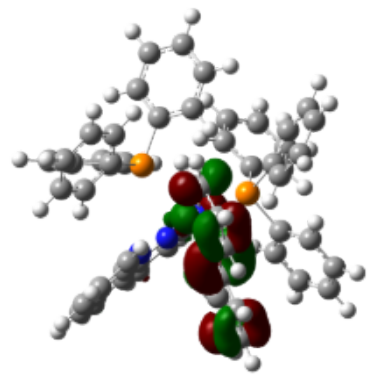 | LLCT (phen→bpy) |
| | | 262 (0.0988) | H-16→L+2 (24%) | 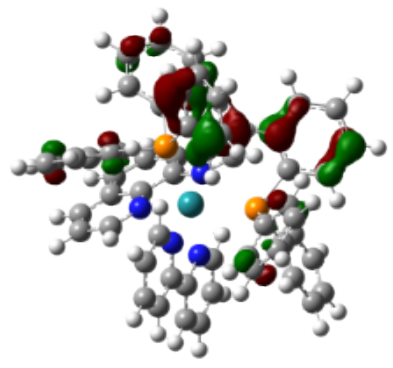 | 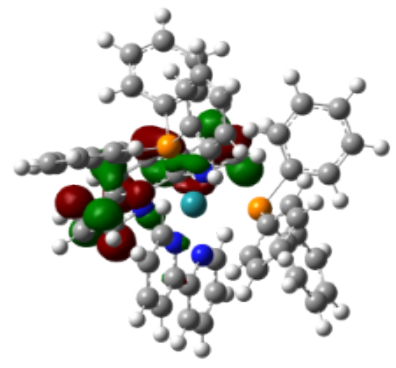 | LLCT (PPh→phen) |

Figure S3 SEM images of (A) pristine SBA-15 at 1 μm; (B) NAT60/SBA-15 at 1 μm; (C) pristine SBA-15 at 5 μm; and (D) NAT60/SBA-15 at 5 μm.

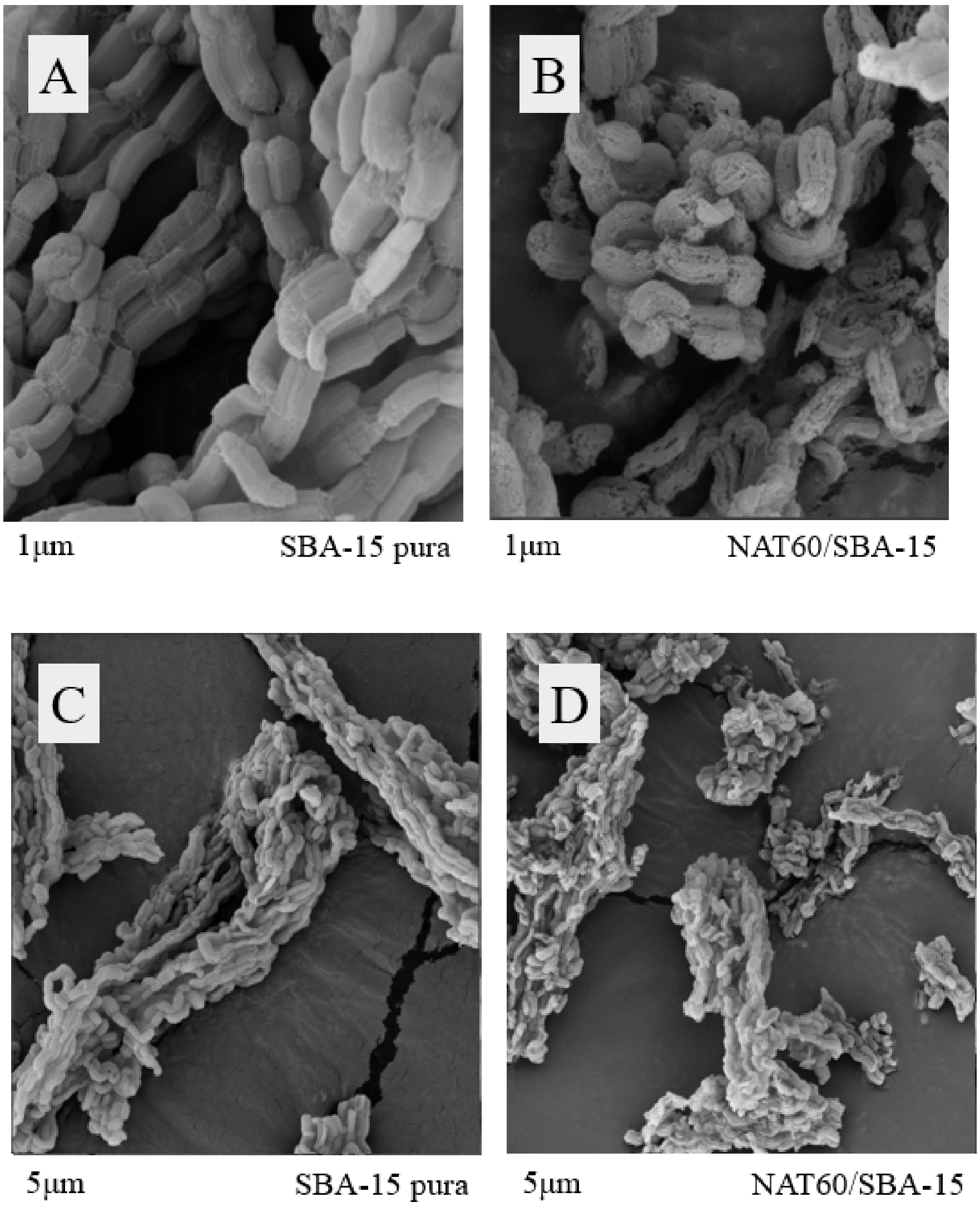
A
1μm
SBA-15 pura
B
1μm
NAT60/SBA-15
C
5μm
SBA-15 pura
D
5μm
NAT60/SBA-15
